# Substrate Recognition Governs Reverse Transcriptase Resistance to Diagnostic Inhibitors in RT-qPCR

**DOI:** 10.3390/diagnostics16121881

**Published:** 2026-06-17

**Authors:** Inês F. Costa, Vânia O. Fernandes, Victor D. Alves, Virgínia M. R. Pires, Joana A. Brás, Pedro Bule, Carlos M. G. A. Fontes

**Affiliations:** 1 NZYtech—Genes & Enzymes, Campus do Lumiar, Building J, 1649-038 Lisbon, Portugal; ines.costa@nzytech.com (I.F.C.); vania.fernandes@nzytech.com (V.O.F.); virginia.pires@nzytech.com (V.M.R.P.); joana.bras@nzytech.com (J.A.B.); carlos.fontes@nzytech.com (C.M.G.A.F.); 2 CIISA—Centre for Interdisciplinary Research in Animal Health, Faculty of Veterinary Medicine, University of Lisbon, 1300-477 Lisbon, Portugal; vdalves@fmv.ulisboa.pt; 3 Associate Laboratory for Animal and Veterinary Sciences (AL4AnimalS), 1300-477 Lisbon, Portugal

**Keywords:** reverse transcriptase, RT-qPCR, inhibitor resistance, molecular diagnostics, substrate recognition, clinical inhibitors, enzyme engineering

## Abstract

**Background:** Reverse transcription is a key step in emerging RNA diagnostics, but reverse transcriptase (RT) enzymes often fail in the presence of inhibitors carried over from clinical samples or introduced during RNA extraction. Here, we dissect the molecular basis of inhibitor resistance in five engineered variants (V1 to V5) of Moloney Murine Leukemia Virus RT, originally optimized for thermostability and catalytic activity. **Methods:** Using a systematic framework that integrates structural analysis, thermal profiling, and diagnostic benchmarking, we evaluated cDNA synthesis from 40 to 70 °C under a panel of 11 clinically relevant inhibitors. **Results:** Across 30 mutations assessed, a recurrent set of substitutions (E69K, E302K/R, W313F, and N454K), present in RT V1 and V4, was associated with enhanced robustness, consistent with strengthened enzyme–nucleic acid engagement, while L435G likely contributes by modulating conformational flexibility. Notably, inhibitor tolerance was maximal at moderate reaction temperatures (≈40 °C), where productive enzyme–substrate interactions best offset inhibitory stress, while the wild-type enzyme was effectively inactivated by several inhibitors under the conditions tested. Although the engineered RTs remained catalytically competent at higher temperatures, increased thermal stress may destabilize productive enzyme–nucleic acid complexes, reducing resistance under inhibitory conditions. **Conclusions:** Together, these findings support substrate engagement as an important determinant of RT robustness and provide practical guidance for engineering inhibitor-resistant RTs for high-sensitivity RT-qPCR.

## 1. Introduction

Quantitative reverse transcription PCR (RT-qPCR) has become a central technology in molecular diagnostics. Moloney Murine Leukemia Virus (M-MuLV) Reverse Transcriptase (RT) plays a fundamental role in the detection of disease-associated RNAs by catalyzing their conversion into complementary DNA (cDNA) [1,2]. M-MuLV RT is a complex, multifunctional enzyme whose catalytic abilities evolved over millions of years of retroviral adaptation to generate a highly effective biocatalyst. It performs three primary functions: (i) RNA-dependent DNA polymerase activity that allows the synthesis of the (−) DNA strand from the viral RNA genome; (ii) RNase H activity that selectively degrades the RNA strand of the resulting RNA-DNA hybrid; and (iii) DNA-dependent DNA polymerase activity, which synthesizes the (+) DNA strand using the (−) DNA strand as a template [3,4,5]. In addition, M-MuLV RT exhibits strand-displacement activity that facilitates cDNA extension across highly structured RNA regions, as well as template-switching ability that supports full-length cDNA synthesis by enabling homology-driven jumps between templates [6,7]. Moreover, M-MuLV also displays a significant error rate, leading to error accumulation over replication cycles, including in the RT gene. The genetic diversity created by these mutations contributes to the adaptability and evolutionary success of retroviruses [8].

**Figure 1:**
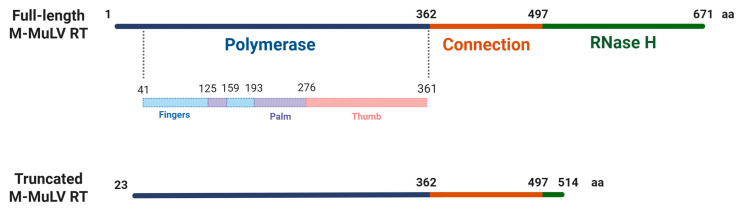
Modular architecture of M-MuLV reverse transcriptase. Schematic representation of the full-length M-MuLV RT, highlighting its distinct domains: the N-terminal DNA polymerase domain, composed of the fingers, palm, and thumb subdomains, the connection domain, and the C-terminal RNase H domain. The truncated RT variant retains only the DNA polymerase and connection domains. This domain organization is responsible for the dual catalytic activity that enables RNA- and DNA-dependent DNA polymerization and the degradation of template RNA in the resulting RNA-cDNA hybrids.

Beyond its virological role, M-MuLV RT is a robust biotechnological tool, serving a variety of applications, including RT-qPCR, RNA sequencing, and the expression profiling of disease-related genes [9]. M-MuLV RT is a modular enzyme composed of two main regions: an N-terminal DNA polymerase domain and a C-terminal RNase H domain (Figure 1). The polymerase module adopts a canonical right-hand structure, found in many other nucleic acid polymerases, with distinct finger, palm, and thumb subdomains that enable the alternative RNA- or DNA-dependent DNA synthesis [10,11]. The RNase H domain degrades the RNA strand of RNA-cDNA hybrids [12]. M-MuLV RT is naturally adapted to operate at moderate temperatures, with optimal activity around 37–42 °C. Above this range, the enzyme loses activity due to thermal inactivation [13]. Moreover, its relatively low processivity often limits the synthesis of long cDNA products, unless assisted through fusion with associated domains or optimized primer-template designs [14,15]. Compared to high-fidelity family B DNA polymerases, M-MuLV RT also has a lower replication accuracy, lacking the 3′-5′ exonuclease proofreading function to ensure error correction [16,17]. To overcome these inherent limitations, multiple studies have focused on engineering M-MuLV RT to improve its thermostability, processivity, and fidelity [15,18,19,20]. While these efforts have significantly expanded RT’s utility in clinical testing, the inherent susceptibility of this enzyme to inhibitory compounds commonly found in crude and semi-crude clinical samples limits the expansion of molecular diagnostics.

Clinical specimens, such as blood, plasma, saliva, swabs, feces, and urine, often contain molecules that impair RT activity and compromise test efficacy [17,21]. Inhibitory agents include complex plant or bacterial polysaccharides (especially in stool), heparin (a known inhibitor of nucleic acid-binding enzymes), and hemoglobin from whole blood, among others [22,23]. In addition, residual chemicals from RNA extraction procedures, such as guanidinium salts, ethanol, or isopropanol, can destabilize the enzyme’s structure and reduce cDNA yield [21]. Thus, despite decades of RT engineering, their resistance to diagnostic inhibitors remains underexplored. Previous engineering efforts have primarily focused on improving M-MuLV RT’s intrinsic properties, such as increasing thermostability through point mutations, deleting or inactivating domains (e.g., by mutating RNase H catalytic residues) to reduce non-specific degradation of the template, and fusing the RT to nucleic acid-binding proteins to improve template affinity and processivity [15,17,18,19,20,24]. Far fewer studies have attempted to increase RT resistance to RT-qPCR inhibitors, and have primarily focused on blood and tissue lysates, with limited scope. One of the few examples describes a thermostable M-MuLV RT pentaplex mutant (E69K, E302R, W313F, L435G, N454K) with increased tolerance to guanidinium salts, ethanol, and acidic polysaccharides [21]. While this suggests that targeted mutations can enhance inhibitor resistance, the molecular mechanisms underpinning inhibitor resistance remain poorly understood, particularly regarding how enzyme structure, surface charge distribution, and thermostability influence susceptibility to inhibition [21].

As the demand for resilient RT-qPCR diagnostics in complex sample matrices continues to grow, a deeper mechanistic understanding of RT–inhibitor interactions is urgently needed. In this study, we systematically characterize five engineered M-MuLV RT variants through a multidisciplinary approach integrating biochemical assays, structural analysis, and diagnostic benchmarking. In addition to comparing engineered RT variants across temperature and inhibitor conditions, we assessed the contribution of enzyme architecture in two complementary ways: first, by comparing full-length and truncated constructs during thermal performance screening, and subsequently, through a focused RT V1 domain/reversion analysis under representative inhibitory conditions. This work provides a systematic evaluation of how discrete molecular features shape the performance of M-MuLV RT in the presence of clinically relevant inhibitors, with direct implications for the rational design of inhibitor-resistant RTs for robust, high-sensitivity RNA diagnostics.

## 2. Materials and Methods

### 2.1. Protein Alignment

Multiple sequence alignment of M-MuLV RT primary sequences was performed using the MAFFT (Multiple Alignment using Fast Fourier Transform) version 7.511.25 (Osaka University, Osaka, Japan) [25,26]. This algorithm is used to identify homologous regions in sequences by analyzing their frequency characteristics after converting the amino acid sequence to volume and polarity values [25]. The resulting protein alignments were used as input for the ESPript 3.0 software (Lyon University, Lyon, France) to visualize the alignment along with the secondary structure of M-MuLV wild-type (WT) RT [27]. The secondary structure of M-MuLV WT RT (PDB: 4MH8) was overlaid in the ESPript alignment to provide a structural context for the analysis of conserved and variable regions [10,28].

### 2.2. Gene Synthesis, Cloning, Expression, and Purification of M-MuLV Variants

M-MuLV RT and mutant derivatives were recombinantly produced and purified from *Escherichia coli* (*E. coli*). RT genes were designed by back-translating protein sequences and optimizing codon usage for expression in *E. coli* to ensure a Codon Adaptation Index (CAI) value higher than 0.8, using the ATGenium codon optimization algorithm (NZYtech, Lisbon, Portugal). Genes were synthesized and directly cloned into pHTP1 expression vector using the NZYEasy cloning & expression kit I (NZYtech) as described previously [29]. Site-directed mutagenesis of the RT V1 was performed using the NZY Supreme Mutagenesis kit (NZYtech) to generate the RT V1 reversion variants: RT V1_G435L (reversion of L435G), RT V1_K454N (reversion of N454K), and the double revertant RT V1_G435L/K454N. The following primers were used: 5′ GGT GAT TCT GGC ACC GCA CGC CGT GGA AGC AC 3′ (G435L forward) and 5′ GCG GTG CCA GAA TCA CCA GCG GCT GAC CCA TG 3′ (G435L reverse), and 5′ GCT GAG CAA CGC CCG TAT GAC GCA TTA TCA GGC ACT G 3′ (K454N forward) and 5′ TAC GGG CGT TGC TCA GCC AGC GAT CTG GCG GCT GTT T 3′ (K454N reverse). Positive clones were fully sequenced in both directions to ensure 100% identity with the designed gene sequences.

The recombinant proteins were expressed in *E. coli* BL21 (DE3). Cells were cultured in 1 L of LB medium supplemented with 0.05 ng/mL kanamycin (NZYtech) and incubated at 37 °C until reaching an OD_600_ between 0.55 and 0.6. Cultures were then incubated on ice for 30 min to slow down the growth rate. Recombinant gene expression was induced by adding 1 mM IPTG (NZYtech), and cells were further incubated at 16 °C for 18 h. Subsequently, harvested cells were lysed with 30 mL NZY Bacterial Cell Lysis Buffer (NZYtech) supplemented with 0.2 µg/mL DNase I, 100 µg/mL Lysozyme, 2.5 mM MgSO_4,_ and 0.5 mM CaCl_2_. Recombinant proteins were purified by immobilized metal ion-affinity chromatography (IMAC) using His GraviTrap™ columns (Cytiva, Wilmington, DE, USA) [30]. The purification protocol comprised a three-step washing protocol with Buffer A (50 mM Na_2_HPO_4_, pH 7.5, 1 M NaCl, 60 mM Imidazole), incorporating an intermediate step in which Buffer A was supplemented with 1% (*v*/*v*) Tween^®^20 to enhance the removal of residual impurities. Finally, recombinant proteins were eluted with 750 µL of Elution Buffer (50 mM Na_2_HPO_4_, pH 7.5, 1 M NaCl, 300 mM Imidazole). All purified proteins were quantified using NanoDrop™ One Microvolume UV-Vis Spectrophotometer (Thermo Scientific, Waltham, MA, USA) and diluted to 1.4 mg/mL with Elution Buffer. For long-term storage, recombinant proteins were diluted to 0.7 mg/mL with glycerol and supplemented with 1 mM DTT (NZYtech) and 0.01% Triton X-100 (Sigma-Aldrich, St. Louis, MO, USA). Recombinant protein solubility, integrity, and purity were assessed by polyacrylamide gel electrophoresis (SDS-PAGE).

### 2.3. Isolation of Mouse Liver Total RNA

Mouse total RNA was extracted from liver tissue, selected for its high RNA content and transcript diversity, providing a suitable matrix to assess RT performance. Extraction was performed using NZYol reagent (NZYtech) according to the manufacturer’s protocol. Briefly, tissue homogenates were prepared in NZYol, followed by phase separation with chloroform. The aqueous phase was recovered, and RNA was precipitated with isopropanol, washed with 75% ethanol, and air-dried. To ensure high purity and remove residual contaminants, the resulting RNA pellet was resuspended in RNase-free water and further purified using the NZY Total RNA Purification Kit (NZYtech), which includes silica membrane-based spin columns. The purified RNA was quantified spectrophotometrically and stored at −80 °C until use.

### 2.4. cDNA Synthesis

Reverse transcription activity was assessed using two-step RT-qPCR. First-strand cDNA was synthesized in a 10 µL RNA mixture containing 5 mM oligo(dT)_18_ primer mix (NZYtech), 1 mM dNTPs (NZYtech), and varying amounts of mouse liver total RNA (1 µg to 10 pg), then incubated at 65 °C for 5 min to facilitate primer annealing to the template. After incubation, the mixture was cooled on ice for 2 min to prevent non-specific annealing. In addition, a 10 µL reverse transcription master mix composed of 2× Reaction buffer for Reverse Transcriptases (NZYtech) and 40 U of NZY Ribonuclease Inhibitor (NZYtech) was added to the primed RNA mix, resulting in a final reaction volume of 20 μL. Unless otherwise indicated, reverse transcription reactions were performed with 0.7 µg of purified RT per 20 µL reaction at different temperatures (40–70 °C). This corresponds to closely comparable molar inputs for the full-length RT variants, which have similar molecular weights, but to a higher molar input for truncated derivatives due to their lower molecular mass. Because the main inhibitor-resistance comparisons were performed among full-length enzymes, enzyme input was considered comparable for the principal benchmarking experiments. Reactions were incubated in a T100 thermal cycler (Bio-Rad Laboratories, Inc., Hercules, CA, USA) for 30 min. Reverse transcription was followed by enzyme inactivation at 85 °C for 5 min. Finally, to degrade the RNA strand from RNA–cDNA hybrids, the cDNA products were treated with RNase H (NZYtech) at 37 °C for 10 min. A standard curve was generated using six serial dilutions starting from 1 µg to calculate the reverse transcription linearity as described above.

To evaluate the impact of the priming strategy, cDNA synthesis was performed as described above, using either 5 µM oligo(dT)_18_ or 2.5 µM random hexamer primer mix (NZYtech). When random hexamers were used, the primer annealing step was carried out at 25 °C for 10 min before reverse transcription. cDNA synthesis was conducted under the same conditions to evaluate RT resistance to RT-qPCR inhibitors, with 5 µL of each inhibitor (4× concentrated) added to the reaction mix. In parallel, control reactions were prepared by replacing the inhibitor with 5 µL of nuclease-free water. All the experiments assessing RT’s resistance to inhibitors used 1 µg of mouse liver total RNA as a template.

### 2.5. Quantitative PCR Analysis

Synthesized cDNA was quantified through real-time qPCR using the Applied Biosystems StepOnePlus™ Real-Time System (Applied Biosystems, Thermo Fisher Scientific, Waltham, MA, USA). qPCR and RT-qPCR experiments were reported in accordance with the general principles of the MIQE Guidelines, including disclosure of primer/probe sequences, reaction conditions, controls, amplification chemistry, replicate structure, and CT-based analysis criteria [31]. The qPCR reactions were carried out in a total volume of 20 µL containing 1× NZYSupreme qPCR Probe Master Mix, ROX Plus (NZYtech, MB43901) or 1× NZYSupreme qPCR Probe Master Mix (NZYtech, MB41601), 4 µM of each primer (forward primer 5′-TCC AAG ATC AAG TCC TTT GTG A-3′ and reverse primer 5′-GTC CCT GAA CAC ATC CTT GT-3′) and 1.25 µM of TaqMan probe (5′/56-FAM/ACT ACA ACC/ZEN/ACC TCA TGC CCA CAA/3IABkFQ/3′) for *Mus musculus* ribosomal protein L27 (*Rpl27*) housekeeping gene (GenBank: NM_011289.3) amplification, using 2 µL of cDNA product as the template (comprising 10% of the total qPCR reaction). The following PCR protocol was used: 95 °C for 2 min followed by 40 cycles of 95 °C for 10 s, and 60 °C for 30 s with measurement of fluorescent signals at the FAM™ channel. A negative control containing water instead of the sample was included in all experiments to rule out contamination of the master mix and primer–probe mix. The linearity of cDNA synthesis for each RT was assessed using a standard curve with RNA concentrations ranging from 1 µg to 10 pg of mouse liver total RNA. The qPCR efficiency was calculated using StepOne Software v2.3 (Applied Biosystems). The inhibition of reverse transcription by each compound in the inhibitor panel was quantified by calculating ΔCT, defined as the difference between the cycle threshold (CT) of the reaction containing the inhibitor and that of the matched no-inhibitor control. A ΔCT value of 1.0 was defined as the threshold, where values below 1 represent no significant inhibition. The ΔCT = 1.0 threshold was selected as a conservative operational cutoff because it exceeded the technical variability observed in matched inhibitor-free control reactions. Across inhibitor-free controls, CT values from technical triplicates showed low dispersion (mean SD = 0.122 cycles; maximum SD = 0.796 cycles), supporting the use of a one-cycle shift as a diagnostically meaningful delay in cDNA synthesis. Quantitative data are presented as mean ± SD from triplicate reactions, as indicated in each figure legend.

### 2.6. PCR Inhibitor Validation

The inhibitor panel used in this study included the following contaminants commonly found in diagnostic matrices: ethanol (PanReac AppliChem, Darmstadt, Germany), isopropanol (PanReac AppliChem), guanidine hydrochloride (Molekula Group, Darlington, UK), NZYol (RNA extraction reagent, NZYtech), sodium citrate tribasic dihydrate (Sigma-Aldrich), heparin (Sigma-Aldrich), animal blood containing potassium ethylenediaminetetraacetic acid (K-EDTA) (LAMPIRE Biological Laboratories, Thermo Fisher Scientific, Waltham, MA, USA), human plasma (Sigma-Aldrich), human urine (Innovative Research, Inc., Novi, MI, USA), human saliva (Innovative Research), and bile salts (Sigma-Aldrich). The concentrations tested for each compound are detailed in the corresponding experimental sections.

The use of inhibitors in both cDNA synthesis and subsequent real-time qPCR quantification was validated using two different assays. (i) To assess the impact of the maximum defined concentration of each inhibitor on the qPCR reaction, 2 µL of a cDNA reaction without RNA and in the presence of each inhibitor (to add the exact inhibitor concentration to the subsequent qPCR mimicking the conditions of a real cDNA reaction) and 2 µL of control cDNA (with no inhibitor, to have cDNA template synthesized without any impact of inhibitors) synthesized from 0.1 ng of RNA were used as templates for a standard qPCR reaction. This setup ensured that any observed inhibition originated from interference with qPCR components rather than cDNA synthesis efficiency. (ii) To exclude possible RNA or DNA contamination from the PCR inhibitors, no template cDNA reactions and negative control cDNA reactions (without RT and template) for each inhibitor were amplified by qPCR, respectively.

### 2.7. Nuclease Detection Assay

To assess the potential presence of RNase or DNase contaminants in the inhibitor panel, two complementary assays were performed. For the detection of RNase activity, 20 µL reactions containing 1 µg of mouse liver total RNA in 1× reaction buffer A (67 mM Tris-HCl, 16 mM (NH_4_)_2_SO_4_, 20 mM KCl, 0.01% (*v*/*v*) Tween^®^ 20, 0.01% (*v*/*v*) Triton X-100) were incubated with each inhibitor (at its maximum working concentration) for 20 min at 37 °C. Reactions were performed in the absence and presence of 40 U of NZY Ribonuclease Inhibitor (RI) (NZYtech). As controls for RNA degradation, we performed a negative control (no-inhibitor control) containing only RNA and a positive control with RNA and 5 ng of RNase A (NZYtech). These were included under identical reaction conditions, with and without RI supplementation, to evaluate its protective effect. After incubation, RNA integrity was assessed by one-step RT-qPCR targeting the *Mus musculus Rpl27* housekeeping gene. The 20 μL reaction mixture contained 1× NZYSupreme One-step RT-qPCR Probe Master Mix (2×) (NZYtech), 4 µM of each primer, 1.25 µM of TaqMan probe, and 5 μL of the incubated RNA sample. To further evaluate RNase activity in the context of the validated two-step RT-qPCR workflow, standard cDNA synthesis reactions were performed using 0.1 ng of mouse liver total RNA as the template, in the absence and presence of 40 U of NZY Ribonuclease Inhibitor (RI), and supplemented with each inhibitor at its maximum concentration. Synthesized cDNA was subsequently quantified by qPCR as described in Section 2.5. RNase activity was assessed by calculating the ΔCT value (CT of the reaction containing the inhibitor minus the CT of the no-inhibitor control), with higher ΔCT values indicating partial or complete RNA degradation. For the detection of DNase activity in the inhibitor panel, the same 20 min incubation at 37 °C was performed using 0.01 ng of pre-synthesized cDNA as the template, instead of RNA. After incubation with each inhibitor, reactions were analyzed directly by qPCR. A reaction containing 0.2 mg/mL of DNase I was used as a positive control for cDNA degradation. The negative control for cDNA degradation consisted of a similar incubation with only the cDNA template (no inhibitor). DNase activity was also evaluated by ΔCT analysis (CT of the reaction containing the inhibitor minus the CT of the no-inhibitor control), with higher ΔCT values indicating greater cDNA template degradation. For a more comprehensive analysis, we compared these results with those obtained in Section 2.6 to discard high ΔCT values resulting from qPCR inhibition rather than DNase activity. Quantitative data are presented as mean ± SD from triplicate reactions, as indicated in each figure legend.

### 2.8. Direct One-Step RT-qPCR on Spiked Saliva Samples Compared with Matched Extracted RNA

Saliva from healthy adult volunteers confirmed as SARS-CoV-2-negative was pooled into four independent samples after treatment with RNase inhibitor (0.02 mg/mL, NZYTech) and heat inactivation (95 °C, 10 min). Each pool was spiked with heat-inactivated SARS-CoV-2 from characterized clinical samples across a broad viral concentration range (100 to 10^6^ copies/µL). This dilution series was designed to span the analytical range of the assay and to assess matrix effects across clinically relevant viral inputs. Spiked samples were analyzed in parallel by direct one-step RT-qPCR and by one-step RT-qPCR following RNA extraction. Non-spiked saliva pools processed under identical conditions served as negative controls. RNA was extracted from an equivalent input volume of each spiked saliva sample using the NZY Mag Universal RNA/DNA Isolation Kit, IVD (NZYtech), according to the manufacturer’s instructions [32], and eluted in the same volume of elution buffer to enable direct volumetric comparison between formats. Extracted RNA was either tested immediately or stored at −80 °C until analysis.

Both formats were tested using the SARS-CoV-2 One-Step RT-PCR Kit, RdRp and N target genes, IVD (NZYtech), which simultaneously detects the RdRp and N target genes in a single fluorescence channel, with human RNase P serving as an internal control in a separate channel. In both formats, the master mix supplied with the kit was replaced by an otherwise identical formulation incorporating RT V1. Reactions were performed in a final volume of 20 µL on an Applied Biosystems 7500 Fast Real-Time PCR System (Applied Biosystems), with 5 µL of input material per reaction (25% *v*/*v*), corresponding to either heat-inactivated spiked saliva or extracted RNA eluate. Reverse transcription was performed for 10 min at 40 °C, followed by PCR amplification according to the manufacturer’s cycling protocol. Results were interpreted according to the kit manufacturer’s assay criteria. An internal control was included in each reaction and interpreted according to the kit manufacturer’s criteria [33]. Qualitative agreement between extraction-based and saliva-matrix formats was assessed as positive/negative concordance across all paired measurements [34]. For positive samples, quantitative agreement was evaluated by calculating ΔCT (CT saliva − CT extracted) and summarizing the mean, median, and range. Method agreement was further assessed by linear regression and Bland–Altman analysis to characterize systematic bias and limits of agreement across the assay’s dynamic range [35].

## 3. Results and Discussion

### 3.1. Engineering M-MuLV RT: Selection of Mutant Variants

Based on previous studies [15,18,19,20], we initially selected four M-MuLV RT mutant variants (RTs V1 to V4) that harbor well-characterized mutations known to enhance thermostability, processivity, and catalytic efficiency (Table 1). These variants represent a widely used benchmark set in M-MuLV RT engineering and provide a solid foundation to evaluate the impact of specific amino acid changes on resistance to RT-qPCR inhibitors. Improved enzyme stability, reflecting more compact enzyme structures, may help enzymes tolerate the changes in the reaction environment caused by inhibitors commonly found in clinical samples [21]. Additionally, we designed and engineered a novel fifth variant (RT V5), incorporating putative resistance-enhancing mutations that had not been previously explored. This construct was specifically intended to enhance inhibitor resistance. Table 1 summarizes the selected M-MuLV RT variants, their amino acid substitutions, putative function, and corresponding reference sources.

**Figure 2:**
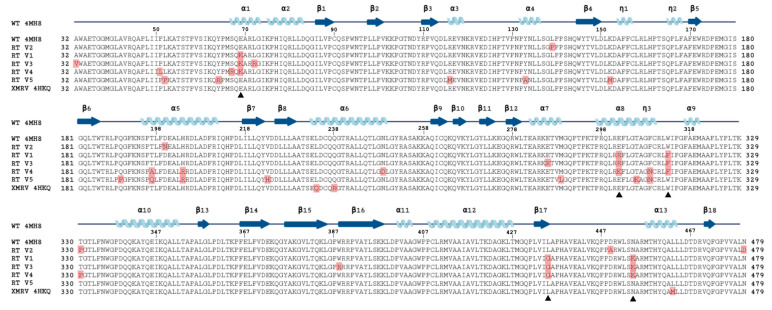
Structural and sequence alignment between M-MuLV RT, its variants V1–V4, and XMRV RT. The alignment was generated using MAFFT and visualized using ESPript 3.0. The secondary structure elements of M-MuLV RT (PDB: 4MH8) are displayed above the alignment, with the mutation sites highlighted in red. α-helices (α) and 3_10_-helices (η) are shown as medium and small light blue squiggles, respectively, while β-strands (β) are represented by dark blue arrows. Relevant common substitutions (E69K, E302K/R, W313F, L435G, and N454K) are indicated by a black arrow. The high sequence identity and structural similarity (r.m.s.d. 2.8 Å over main-chain atoms) support the use of XMRV RT (PDB: 4HKQ) as a structural proxy for analyzing M-MuLV RT mutations.

To gain a deeper mechanistic insight into the role of each specific amino acid substitution, we integrated biochemical data from previous studies with structural analysis of the RNA/DNA-bound RT complex of xenotropic murine leukemia virus-related virus (XMRV), a close homolog of M-MuLV RT [36]. These two RTs share 95.7% sequence identity and 98.2% similarity (Figure 2), with an r.m.s.d. of 2.8 Å for main-chain carbon atoms (when comparing the apo-M-MuLV RT, PDB: 4MH8, with the RNA/DNA-bound XMRV RT complex, PDB: 4HKQ). Given these high structural and sequence similarities, the XMRV RT complex is a reliable structural proxy for functional predictions of M-MuLV RT. Using this structural framework in combination with published biochemical data, we assigned putative functional roles to each mutation (Table S1). Furthermore, an integrated assessment of the accumulated mutations in each M-MuLV RT mutant variant (RTs V1–V5) provided insights about their expected functional properties as summarized in Table 1. Together, this variant set allows us to decouple three variables that are often confounded in RT-qPCR: intrinsic thermostability, domain architecture, and chemical inhibition. We therefore benchmarked cDNA synthesis across temperature and inhibitor space under a standardized ΔCT framework and then tested mechanistic hypotheses using targeted reversions.

### 3.2. Optimized Recombinant Expression and Purification of M-MuLV RT Variants

After selecting the five different M-MuLV RT mutant variants, we proceeded with their recombinant production for experimental evaluation. The M-MuLV WT RT was also included as a reference, bringing the total number of enzymes analyzed in this study to six. The M-MuLV RT enzyme has a modular architecture comprising an N-terminal polymerase domain and a C-terminal RNase H domain. RNA-cDNA hybrids act as stable intermediates for subsequent amplification, while high temperatures during PCR cycling contribute to RNA degradation. Together, these factors render RNase H activity unnecessary for most RT-qPCR applications [2]. However, the presence of an intact, yet catalytically inactivated, RNase H domain may contribute to overall enzyme stability, particularly at elevated temperatures [37,38]. In addition, while some studies report the challenge of producing soluble RT proteins, others have successfully improved this through structural flexibility at the connection domain (L435K, N454K) [39]. Therefore, in this study, we systematically evaluated the influence of both full-length and truncated RT variants by producing two versions of each of the six RT variants: (i) full-length enzymes, and (ii) truncated derivatives containing only the N-terminal polymerase domain. This comparison was designed to determine whether removal of the RNase H domain affected baseline thermal performance and to guide the selection of the most suitable enzyme architecture for subsequent inhibitor-resistance analyses. The genes encoding M-MuLV WT RT and the five engineered mutant variants (RT V1–V5) were designed with codon optimization for *E. coli* expression, synthesized, and cloned into a high-yield expression vector under the control of a T7 promoter. In general, the full-length RT variants contained an inactivated C-terminal domain (D524G, E562Q, D583N) to ensure a complete loss of RNase H activity [37], while maintaining the structural integrity of the full-length enzyme (see above, Table 1). These three mutations were the only amino acid changes incorporated into the full-length WT RT. Expression was conducted in *E. coli* BL21 (DE3) cells, and soluble protein production was confirmed for all variants. SDS-PAGE analysis of the 12 recombinant enzymes, comprising six full-length and six truncated variants, confirmed their solubility and molecular weight (Figure S1). A preliminary reverse transcriptase activity assay at 50 °C confirmed that all RT variants retained the capacity for cDNA synthesis, providing a strong foundation for further biochemical characterization.

### 3.3. RNA Priming Strategies: Oligo(dT) vs. Random Hexamers Across a Broad Temperature Spectrum

To specifically investigate the resistance of M-MuLV RT variants to inhibitors independently of other enzymes typically involved in diagnostic workflows (e.g., DNA polymerases, uracil-DNA glycosylases, single-stranded DNA-binding proteins), we assessed cDNA synthesis efficiency using a two-step reverse transcription approach. Accordingly, reverse transcription was first performed under various diagnostic scenarios, and cDNA yield was subsequently quantified via qPCR. Total RNA from mouse liver tissue was used as the input template, since total RNA is typically the template in molecular diagnostics. For viral diagnostics, target-specific priming is common; here we used total RNA to provide a controlled and reproducible benchmarking substrate. Reverse transcription across a broad temperature range (30–70 °C) requires an efficient priming strategy, which may include specific primers, oligo(dT) primers, or random hexamers [40]. In molecular diagnostics, target-specific primers provide high sequence specificity, while oligo(dT) primers selectively target polyadenylated mRNA transcripts, making them broadly applicable to gene expression analysis. Random hexamers, in contrast, enable non-specific priming, facilitating cDNA synthesis from diverse RNA species, including rRNA and viral RNA. However, previous studies suggest that the priming efficiency of random hexamers is temperature-dependent [40], which could potentially affect cDNA yield in high-temperature reverse transcription assays.

To systematically evaluate priming efficiency across different temperatures, we selected the full-length RT V1, a putatively highly thermostable variant with enhanced primer–template affinity [18]. Reverse transcription reactions were performed using increasing RNA input amounts (up to 1 µg per 20 µL reaction) to assess the efficiency of cDNA synthesis across a broad RNA concentration range. To exclude the possibility that differences in priming efficiency, particularly when using random hexamers, were caused by insufficient RT enzyme levels, we tested four different enzyme loads (0.6 to 0.9 µg per 20 µL reaction). The data, summarized in Figure S2, demonstrated that oligo(dT) primers consistently supported efficient cDNA synthesis across all tested temperatures and enzyme concentrations, with reaction slopes and calculated efficiencies falling within the acceptable 85–110% range. In contrast, random hexamers exhibited a significant loss of priming efficiency at 60 °C. At this temperature, anomalously high reaction efficiencies were observed at lower enzyme concentrations (120.4% for 0.6 µg and 119.9% for 0.7 µg), likely reflecting non-linear amplification due to inefficient or unstable priming. Increasing the enzyme concentration to 0.8–0.9 µg normalized efficiencies (100.9–88.3%), suggesting that higher enzyme loads can partially compensate for reduced primer–template affinity [40]. Altogether, these findings support the use of oligo(dT) priming for robust, temperature-tolerant cDNA synthesis in this benchmarking framework, and as such, this priming method was selected for all subsequent experiments.

### 3.4. Impact of Temperature on M-MuLV RT Mutant Performance

**Figure 3:**
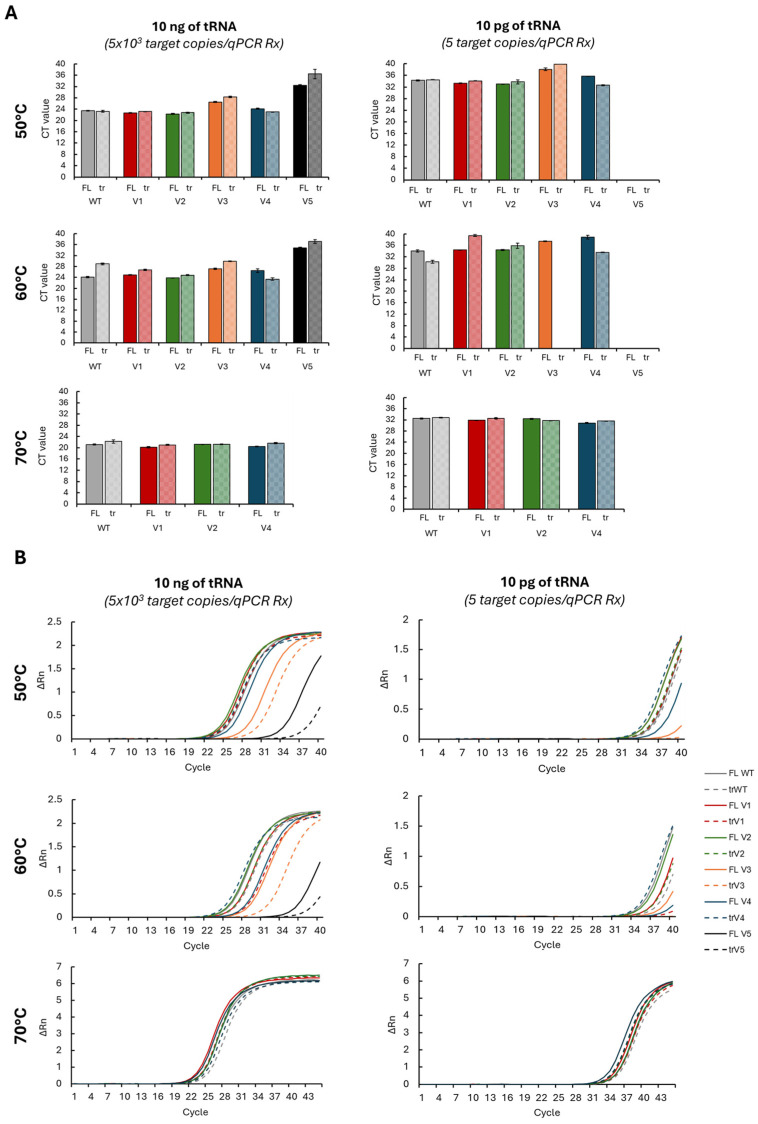
Comparative thermal performance of M-MuLV RT mutant variants across two different RNA input concentrations. (**A**) cDNA synthesis efficiency at three temperatures (50 °C, 60 °C, and 70 °C) using 10 ng and 10 pg of mouse liver total RNA per cDNA synthesis reaction. Reactions were performed with full-length (FL) and truncated (tr) RT variants, and cDNA was quantified by qPCR targeting the *Mus musculus Rpl27* gene. Data are presented as mean ± SD from triplicate reactions. (**B**) Amplification plots of the reactions presented in Panel A. Oligo d(T)_18_ primers were used in all reactions. ΔRn values were normalized using ROX dye as a passive reference. RT V5 demonstrated reduced efficiency in converting 10 ng of total RNA at temperatures of 50 °C and 60 °C, with no activity observed at a low RNA input of 10 pg. RT V3 was excluded from testing at 70 °C due to its reduced performance at 60 °C.

To evaluate the thermal performance of all M-MuLV RT variants, we next assessed cDNA synthesis efficiency at 50 °C, 60 °C, and 70 °C using two concentrations of mouse liver total RNA, 10 ng and 10 pg, corresponding to approximately 5 × 10^4^ and 50 copies of *Mus musculus* Rpl27 mRNA per cDNA synthesis reaction, respectively. These levels reflect clinically relevant target ranges, simulating either typical diagnostic samples or challenging low-copy detection scenarios [19,41]. The results displayed in Figure 3 revealed that, except for RT V5, at 50 °C, all variants supported efficient cDNA synthesis, establishing a baseline functional threshold for M-MuLV RT. However, at 60 °C, RT V3 exhibited reduced activity at the lowest RNA input, indicating compromised thermal stability and/or template affinity. Given this limitation, RT V3 and V5 were excluded from subsequent 70 °C assays. Surprisingly, the WT RT retained functional activity at 70 °C, performing comparably to engineered variants. This challenges the assumption that the WT enzyme is inherently thermolabile. Its preserved function may reflect intrinsic flexibility and/or effects of the full-length construct used here. Additionally, high enzyme-to-template ratios may have compensated for partial thermal inactivation, enabling efficient cDNA synthesis even with limited active enzyme molecules. Importantly, this apparent thermal competence was observed under inhibitor-free conditions and should not be interpreted as equivalent to chemical robustness.

Across tested conditions, full-length (FL) variants consistently outperformed their truncated counterparts. This suggests that the catalytically inactive RNase H domain may enhance structural integrity or reduce aggregation, contributing to stability at elevated temperatures. Based on these results, full-length versions of RT V1, V2, V4, and WT were retained for further analysis. RT V3 was retained for later inhibitor profiling because it carries a distinct mutational architecture that proved informative mechanistically, while RT V5 was excluded due to poor performance. In this study, enzyme input was standardized primarily by protein mass rather than by independently measured specific catalytic activity. This approach supports the principal comparisons among full-length RT variants with comparable molecular weights and is further reinforced by ΔCT normalization to matched no-inhibitor controls for each enzyme (see below). Comparisons involving truncated variants should nevertheless be interpreted with appropriate caution, since equal protein mass corresponds to a higher molar input for these lower-molecular-weight enzymes. Importantly, despite this potential molar advantage, truncated variants showed reduced thermal performance relative to their full-length counterparts, supporting the decision to focus subsequent inhibitor-resistance analyses on full-length RTs.

### 3.5. Linearity of cDNA Synthesis Across a Broad Range of RNA Concentrations

**Figure 4:**
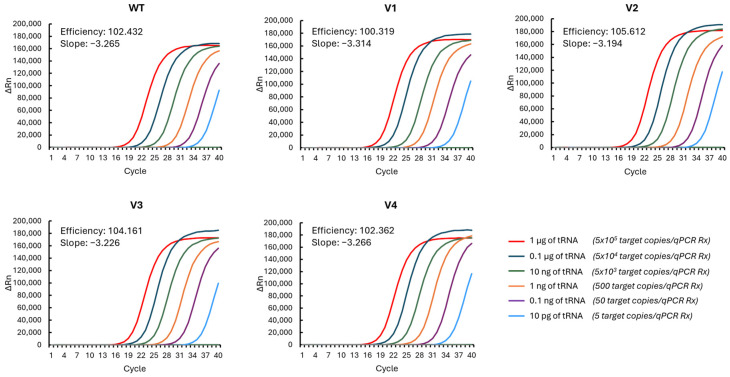
Linearity of cDNA synthesis across a broad RNA input range. Reverse transcription reactions were performed at 50 °C using six RNA input concentrations (1 µg to 10 pg of mouse liver total RNA) for each of the five selected full-length M-MuLV RT variants: WT, RT V1, RT V2, RT V3, and RT V4. Reactions were primed with Oligo d(T)_18_, and cDNA synthesis efficiency was evaluated by qPCR amplification of the *Mus musculus Rpl27* gene. Amplification plots show consistent performance for all enzymes across the entire dynamic range, with no major losses in efficiency at low RNA inputs. RT V1 and RT V4 exhibited the most robust linear behavior, while RT WT and RT V3 displayed slight reductions in amplification sensitivity at the lowest RNA concentrations. All reactions were performed in triplicate.

While the previous experiments focused on two diagnostically relevant RNA input levels, we next aimed to validate the linear quantitative performance and dynamic range of the five full-length selected M-MuLV RT variants across a broad range of RNA concentrations. Assessing cDNA synthesis linearity is essential for molecular diagnostics, where RNA input levels vary significantly across clinical samples [42]. To this end, reverse transcription reactions were performed at 50 °C using 10-fold serial dilutions of mouse liver total RNA, ranging from 1 µg to 10 pg per cDNA synthesis reaction, to generate a six-point standard curve. The quantification of cDNA yield via qPCR, displayed in Figure 4, revealed consistent linearity across all tested concentrations for the five RT variants.

The data indicate that all enzymes display efficient cDNA synthesis across the entire input range, with no significant loss of synthesis efficiency at lower RNA inputs. RT V1 and RT V4 demonstrated the most consistent performance, while WT and RT V3 showed slightly delayed amplification at lower template concentrations, suggesting a lower sensitivity. These results confirm that all five full-length RT variants exhibit high accuracy across a broad input range, validating their suitability for diagnostic workflows requiring precise RNA quantification, including low-copy detection. All five variants were therefore retained for downstream analysis of inhibitor resistance.

### 3.6. Defining a Comprehensive Panel of Clinically Relevant Inhibitors

To evaluate the resistance of RT variants, we developed a panel of 11 clinically relevant inhibitors, including both exogenous contaminants (e.g., guanidinium salts, alcohols, NZYol) and endogenous substances from biological matrices (e.g., plasma, saliva, bile salts) (Table S2). These compounds are known to impair reverse transcription efficiency and can compromise diagnostic assay performance [18,43]. Assays were initially calibrated using RT V1 at 40 °C, with high (1 µg) and low (0.1 ng) RNA input to model typical and limiting detection scenarios. We deliberately selected a moderate temperature to isolate chemical inhibition effects, minimizing temperature-induced confounding. Each inhibitor was tested at four concentrations to define two representative conditions: one permissive (minimal inhibition) and one stringent (strong inhibition) [43,44,45]. We used ΔCT values relative to no-inhibitor controls to quantify the inhibition. A conservative threshold was applied: ΔCT ≤ 1.0 was considered negligible, within the range of typical qPCR variation, while ΔCT > 1.0 indicated operationally or diagnostically meaningful inhibition. The suitability of this cutoff was supported by the low variability in the inhibitor-free control reactions, with technical triplicates showing a mean CT SD of 0.122 cycles and a maximum SD of 0.796 cycles. Therefore, a ΔCT shift of 1.0 cycle was considered to exceed normal assay variation and to represent operationally meaningful inhibition.

Initial results showed that RT V1 retained activity in the presence of multiple inhibitors, including sodium citrate, plasma, EDTA-blood, saliva, urine, and bile salts, even at high concentrations (Figure S3A). In contrast, guanidine hydrochloride and NZYol had strong inhibitory effects, particularly at the highest concentrations. To refine sensitivity thresholds, we expanded the concentration ranges for inhibitors that had no initial effect (Figure S3B). The highest permissible inclusion level was 25% (*v*/*v*) for biological matrices. Based on these extended assays, we selected one permissive and one stringent concentration *per* inhibitor for use in all subsequent tests (see Table S2), thereby establishing a standardized and reproducible framework for assessing inhibitor resistance across RT variants.

### 3.7. Do RT Inhibitors Primarily Affect cDNA Synthesis or Detection?

Having established a panel of clinically relevant inhibitors along with defined concentration ranges, we next sought to determine whether the observed inhibitory effects were specific to reverse transcription or also attributable to interference with the downstream qPCR-based detection. Since cDNA quantification relies on a two-step workflow, with approximately 10% (*v*/*v*) carryover into qPCR, it was essential to distinguish between inhibition affecting the reverse transcription step versus qPCR amplification itself. To address this, we conducted control qPCR reactions in the presence of diluted inhibitor concentrations (equivalent to one-tenth of the original concentrations used during cDNA synthesis), using pre-synthesized cDNA as template. The results, shown in Figure S4, confirmed that most inhibitors did not affect qPCR detection at the tested concentrations. However, the obtained ΔCT of sodium citrate at 2.5 mM was 0.95, which approaches the threshold for detection interference defined in this study, suggesting that it may influence qPCR efficiency at higher concentrations. Accordingly, future analyses involving sodium citrate will be interpreted with caution, as its inhibitory effect may extend beyond reverse transcription and affect qPCR detection.

### 3.8. Inhibitor-Associated Nucleases: Do Common Clinical Matrices Contribute to Nuclease Activity?

In RNA molecular diagnostics, ribonuclease inhibitors (RIs) are routinely added to preserve RNA integrity during sample processing and amplification. However, the presence of RIs can mask the extent of endogenous ribonuclease (RNase) contamination, making it difficult to assess whether biological matrices or chemical additives inherently harbor nuclease activity [46]. More importantly, RI supplementation may not provide full protection, particularly when nucleases are present at high levels or when inhibitors interfere with RI function. To investigate whether the inhibitors used in this study naturally contain RNase activity, we incubated mouse liver total RNA with each inhibitor in the presence or absence of RI and quantified residual RNA via one-step RT-qPCR. Reactions containing a known amount of RNase A (5 ng) served as positive controls for RNA degradation, and inhibitor-free reactions were used to calculate ΔCT values. As shown in Figure S5, RNA integrity was consistently reduced in samples treated with EDTA-blood, plasma, urine, and saliva in the absence of RI, indicating that these clinical matrices harbor endogenous RNases. These findings support two key conclusions: first, that RNase contamination is common in clinical samples such as blood, plasma, urine, and saliva, and second, that supplementing reactions with 40 U of RI per 20 µL is sufficient to prevent RNA degradation in this assay format and under the conditions tested.

While RNA degradation is a well-recognized challenge in reverse transcription, the stability of newly synthesized cDNA in the presence of potential deoxyribonucleases (DNases) has received far less attention. DNase contamination from clinical specimens, such as saliva or blood, may compromise assay performance, particularly in workflows that separate reverse transcription from qPCR detection. To explore this possibility, we incubated 0.01 ng of pre-synthesized cDNA with each inhibitor at 37 °C for 20 min and then quantified cDNA levels by qPCR. To distinguish DNase effects from qPCR inhibition, we included the results obtained in Section 3.7. As shown in Figure S6, sodium citrate and blood-EDTA produced a comparable ΔCT with that obtained from qPCR inhibition. In contrast, saliva produced a higher ΔCT increase, suggesting that this inhibitor may contain mesophilic DNases active at 37 °C but inactive at higher temperatures, such as the 50 °C used for cDNA synthesis. These findings highlight the need to evaluate both RNA- and DNA-degrading enzyme activity during diagnostic assay validation, especially in two-step RT-qPCR protocols. Finally, we verified that none of the inhibitor stocks produced detectable amplification in the absence of RNA or cDNA templates, confirming that there was no cross-reactivity or nucleic acid contamination in the reagents.

### 3.9. Comparative Inhibitor Resistance Among Full-Length M-MuLV RT Variants

**Figure 5:**
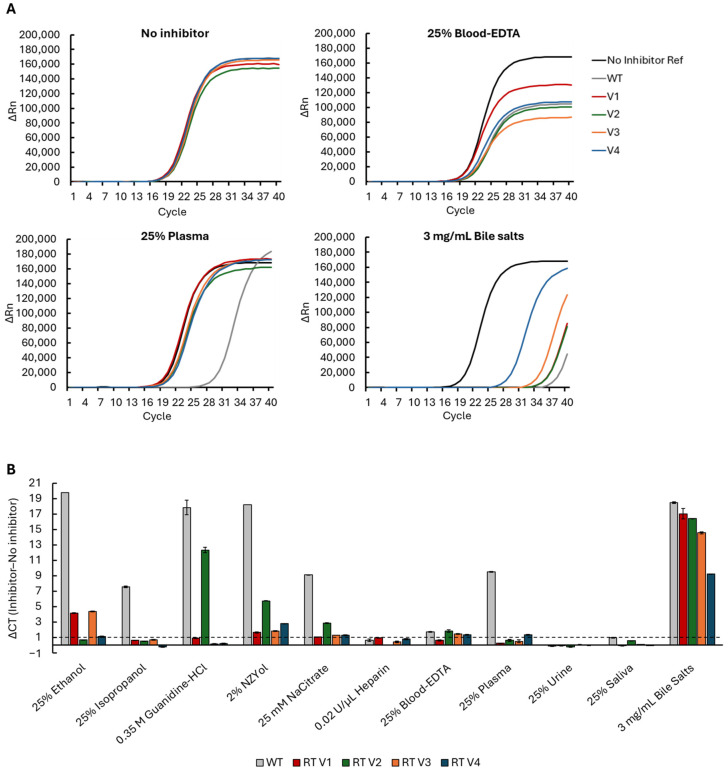
Comparative resistance of full-length M-MuLV RT variants to clinically relevant inhibitors at 40 °C. (**A**) Representative amplification curves showing cDNA synthesis by the wild-type (WT) and engineered RT variants (V1–V4) in the absence of inhibitors and the presence of 25% (*v*/*v*) blood-EDTA, 25% (*v*/*v*) plasma, or 3 mg/mL bile salts. Fluorescence was monitored during qPCR amplification of Mus musculus Rpl27 cDNA. (**B**) ΔCT values for each RT enzyme calculated as the difference in cycle threshold (CT) between reactions performed with and without the indicated inhibitors, using the highest tested concentration (see Section 3.6). Lower ΔCT values indicate higher resistance to inhibition. The horizontal dashed line marks the defined inhibition threshold (ΔCT = 1.0). While most inhibitors primarily delayed amplification (increased CT), blood-EDTA also reduced fluorescence intensity (panel **A**), suggesting additional interference with fluorescence detection. A magnified view of the lower ΔCT range is provided in Figure S7 to facilitate comparison among engineered RT variants while preserving the full dynamic range of inhibition in (panel **B**). Data in (panel **B**) are presented as mean ± SD from triplicate reactions.

To assess inhibitor resistance independently of thermal stress, we performed initial cDNA synthesis assays at 40 °C using 1 µg of mouse liver total RNA under standardized reaction conditions. This temperature allowed for a controlled comparison of full-length WT and engineered M-MuLV RT variants (V1–V4) challenged with a panel of clinically relevant inhibitors, with chemical resilience as the primary variable. For each enzyme, reactions were conducted in the absence and presence of two inhibitor concentrations (as defined in Section 3.6), and ΔCT values were calculated relative to inhibitor-free controls. The data, presented in Figure 5, using the highest inhibitor concentrations, revealed that while all five enzymes performed similarly under inhibitor-free conditions (Figure 5A), the WT enzyme displayed markedly reduced resistance to several inhibitors, including ethanol, isopropanol, guanidine hydrochloride, NZYol, sodium citrate, and plasma (Figure 5B). In contrast, RT variants, particularly V1, V3, and V4, consistently maintained low ΔCT values across most conditions, reflecting superior inhibitor tolerance (Figure 5B and Figure S7). Notably, RT V2 also demonstrated robust performance under stress but exhibited occasional sensitivity to guanidine hydrochloride and NZYol, suggesting marginally reduced resilience under denaturing conditions. These findings indicate that WT RT can retain measurable activity under clean reaction conditions but remains highly vulnerable to chemical perturbation. Thus, inhibitor resistance cannot be inferred from thermal activity alone, supporting the view that thermal competence and chemical robustness are related but distinct RT properties. By contrast, at 40 °C, 0.02 U/μL heparin and 25% (*v*/*v*) urine or saliva had negligible effects on all RTs tested, including the WT, indicating that these matrices do not interfere with RT performance at conventional reaction temperatures. None of the enzymes, including the engineered variants, retained full activity in the presence of high concentrations of bile salts (Figure 5A,B), underscoring the particularly disruptive effect of this inhibitor on enzymatic activity and/or potentially on nucleic acid recognition/stability.

Taken together, these findings reveal the limitations of non-engineered RTs when challenged with partially purified or inhibitor-rich clinical samples. In contrast, the data underscore the strong and consistent inhibitor resistance of RT V1, V3, and V4, confirming the initial hypothesis that rational engineering strategies aimed at improving thermostability will also impact inhibitor resilience. While most inhibitors primarily delayed amplification (reflected in higher CT values), blood-EDTA also caused a modest but detectable reduction in fluorescence signal (Figure 5A), suggesting additional interference with signal-detection mechanisms. Although all engineered RTs showed improved resistance profiles, RT V2 exhibited some vulnerability under highly denaturing conditions.

### 3.10. Influence of cDNA Synthesis Temperature on RT Inhibitor Resistance

**Figure 6:**
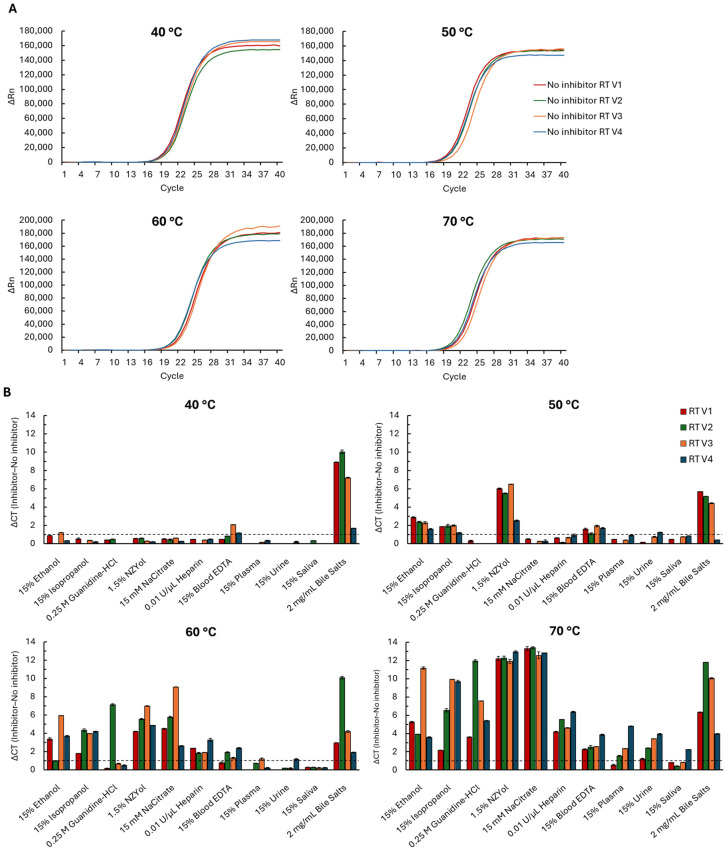
Temperature-dependent inhibitor resistance of engineered thermostable RT variants. (**A**) Real-time amplification curves obtained at 40 °C, 50 °C, 60 °C, and 70 °C in the absence of inhibitors demonstrate that all four thermostable RT variants (RT V1–V4) exhibit consistent performance across the full temperature range, confirming their robust intrinsic thermostability. (**B**) ΔCT values for each RT variant at increasing cDNA synthesis temperatures (40 °C to 70 °C) in the presence of the lowest tested concentration of each inhibitor. At 50 °C, a modest decline in resistance became apparent for several inhibitors, particularly ethanol, isopropanol, NZYol, and blood-EDTA. As temperature increases to 60 °C and 70 °C, widespread reductions in resistance are observed, with nearly all inhibitors causing elevated ΔCT values, indicating compromised RT activity. Notably, bile salts showed reduced inhibitory effects at higher temperatures (≥50 °C), potentially due to thermally induced micellar disassembly. In contrast, saliva maintained low inhibitory effects across all temperatures. These findings reveal a trade-off between elevated reaction temperatures and inhibitor tolerance, supporting 40 °C as the optimal synthesis temperature for diagnostic RT-qPCR applications involving partially purified or crude samples. Data in panel B are presented as mean ± SD from triplicate reactions.

In molecular diagnostics, elevated reaction temperatures are widely employed to improve cDNA synthesis efficiency by reducing RNA secondary structures and non-specific priming [14]. Given that thermostability was a central design goal in engineering RT variants V1 through V4, we hypothesized that their resistance to inhibitors might be sustained, or even improved, at higher cDNA synthesis temperatures. To test this, we conducted two-step RT-qPCR assays at 50 °C, 60 °C, and 70 °C using the same inhibitor panel and ΔCT analysis described in Section 3.9. The results, displayed in Figure 6 (lowest inhibitor concentration) and Figure S8 (highest inhibitor concentration), revealed that all four RT variants performed similarly across the full 40–70 °C range in the absence of inhibitors (Figure 6A and Figure S7A), confirming their intrinsic thermostability. However, their inhibitor resistance declined sharply with increasing temperature (Figure 6B and Figure S7B). At the highest inhibitor concentrations, raising the reaction temperature from 40 °C to 50 °C led to a sudden and widespread loss of tolerance (typically ΔCT > 1), particularly in the presence of ethanol, isopropanol, and NZYol (Figure S8). This precluded a reliable assessment of inhibitor effects at 60 °C and 70 °C under these conditions. Therefore, subsequent evaluations at these temperatures were performed using the lowest inhibitor concentrations (Figure 6B). Even at lower concentrations, the onset of temperature-induced sensitivity was evident. At 50 °C, inhibitors that had little to no effect at 40 °C, such as ethanol, isopropanol, NZYol, and blood-EDTA, began to impair performance across all variants. At 60 °C, this effect became more pronounced, with a widespread increase in ΔCT values, and by 70 °C, cDNA synthesis was severely compromised for all RTs tested. Overall, RT V1 and RT V4 retained the lowest ΔCT values as temperature increased, although the rank order between engineered variants depended on inhibitor class.

These findings show that, although the engineered RTs remain catalytically competent at elevated temperatures in inhibitor-free reactions, the combination of thermal and chemical stress sharply reduces inhibitor tolerance. This pattern is consistent with a model in which higher reaction temperatures may partially destabilize productive enzyme–nucleic acid complexes and/or increase the vulnerability of RTs to chemically induced perturbations. Direct measurements of substrate binding or conformational dynamics across temperature were not performed; therefore, this interpretation should be considered a mechanistic hypothesis supported by the observed functional trends. Together, these data distinguish thermal catalytic competence from inhibitor resilience. Although WT and engineered RTs can support cDNA synthesis under selected inhibitor-free conditions, the combination of thermal and chemical stress imposes additional constraints beyond those predicted by thermal performance alone. Interestingly, bile salts exhibited an opposite trend: inhibition diminished at higher temperatures, possibly due to micelle disassembly that reduces protein sequestration and/or disruption of enzyme surfaces [47]. In addition, saliva was the only inhibitor for which no temperature-dependent variation was observed, with consistently low inhibition across all conditions tested. Together, these results highlight a trade-off in diagnostic workflows: elevated temperatures can improve RT specificity, but may compromise robustness in the presence of inhibitors. In this study, inhibitor tolerance was consistently maximal at 40 °C, suggesting that moderate synthesis temperatures may offer a more reliable operational window for RT-qPCR in inhibitor-rich or partially purified matrices. Notwithstanding this general trend, some matrices, including 15% urine, saliva, and plasma, had a limited impact on RT V1 performance across 40–70 °C, indicating that certain inhibitors remain manageable even at elevated temperature, depending on enzyme design.

### 3.11. Direct Detection of SARS-CoV-2 in Non-Extracted Saliva Using RT V1 in One-Step RT-qPCR

Having defined inhibitor tolerance under controlled benchmarking conditions, we next evaluated whether RT V1 preserves performance in an extraction-free workflow using a clinically relevant matrix. We therefore compared one-step RT-qPCR performed directly on saliva with matched testing of RNA extracted from the same samples. This experiment was designed as a controlled proof-of-concept comparison between direct and extraction-based workflows, rather than as a full clinical validation study. Donor-negative saliva was combined into four independent pools, each spiked with heat-inactivated SARS-CoV-2 derived from characterized clinical positive samples across a broad viral input range (100 to 10^6^ copies/µL). For both formats, 5 µL of input was added per 20 µL reaction (25% *v*/*v*), corresponding to non-extracted spiked saliva or extracted RNA eluate. RNA extraction was performed from an equivalent input volume, and the eluate was collected in the same volume to enable direct volumetric comparison between formats. Detection was performed using the SARS-CoV-2 One-Step RT-PCR Kit, RdRp and N target genes, IVD primer–probe mix, which targets RdRp and N genes in the same fluorescence channel, with human RNase P as an internal control in a separate channel. In both formats, the original master mix was replaced by an otherwise identical formulation incorporating RT V1.

**Figure 7:**
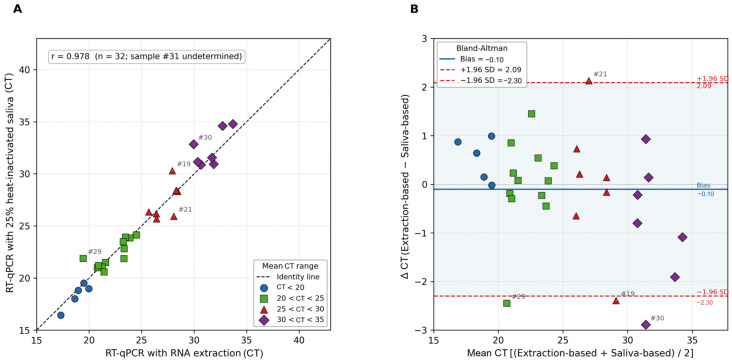
Agreement between SARS-CoV-2 CT values obtained from direct saliva testing and matched extraction-based testing using an RT V1-based one-step RT-qPCR workflow. (**A**) Scatter plot of CT values for spiked saliva samples tested directly (25% *v*/*v* heat-inactivated saliva input) versus CT values obtained after RNA extraction from the matched saliva sample; the dashed line indicates the identity line. (**B**) Bland–Altman plot showing ΔCT (extraction-based − saliva-based) versus the mean CT of the two methods, with the bias and 95% limits of agreement indicated.

Across 32 paired spiked-saliva measurements (direct versus extracted), 31 valid pairs were obtained since sample #31 was undetermined in the extraction-based format (Table S3). CT values obtained in the presence of saliva closely matched those obtained from the paired extracted samples (mean CT: saliva 25.06 vs. extracted 24.96), with negligible systematic bias (mean ΔCT saliva–extracted = +0.10 cycles; median = −0.08 cycles; range −2.13 to +2.89; Table S3). Linear regression confirmed close agreement across the dynamic range (r = 0.978; *n* = 31; Figure 7A), and Bland–Altman analysis showed a small bias of −0.10 cycles for ΔCT extracted saliva (limits of agreement −2.30 to +2.09; Figure 7B), consistent with the ΔCT sign convention above. In addition, specificity was assessed using 20 independent SARS-CoV-2-negative clinical saliva samples, which were tested in parallel in both formats (direct versus extracted). All 20 samples were classified as negative in both the direct and extraction-based workflows (Table S3). Together, these results show that RT V1 supports one-step RT-qPCR directly in saliva at 25% (*v*/*v*) input, across a broad viral concentration range, without detectable loss of agreement relative to matched extraction-based testing under the conditions evaluated. This experiment should be interpreted as a controlled proof-of-concept matrix comparison rather than a clinical validation study; further testing in more inhibitory matrices, such as whole blood or feces, will be important to define the broader applicability of this approach.

### 3.12. Molecular Determinants of Inhibitor Resistance

**Figure 8:**
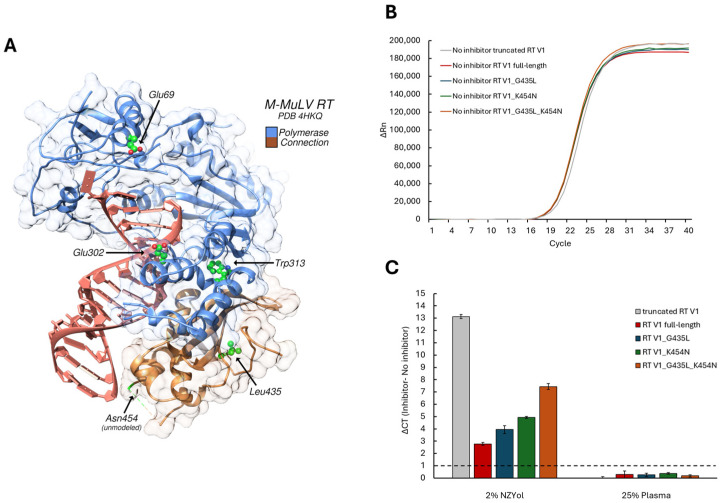
Structural mapping and functional impact of inhibitor-resistance mutations in engineered M-MuLV RTs. (**A**) Structural model of the RNA/DNA-bound XMRV RT complex used as a proxy for M-MuLV RT (PDB ID: 4HKQ). Two main domains are highlighted: the polymerase domain (blue) and the connection domain (brown). A bound RNA–DNA hybrid is shown in pink. The five amino acid substitutions identified in RT variants V1, V3, and V4, Glu69, Glu302, Trp313, Leu435, and Asn454, are indicated as space-filling models and correspond to the mutations associated with enhanced resistance to diagnostic inhibitors. (**B**) Real-time amplification curves obtained in the absence of inhibitors demonstrate that all three RT V1 reversion variants and the RNase H-inactive truncated version exhibit performance similar to that of RT V1. (**C**) Functional impact of reverting flexibility-associated mutations L435G and N454K in RT V1. ΔCT values were measured in two-step RT-qPCR at 40 °C using 1 μg of mouse liver RNA in the presence of NZYol and plasma, at the highest concentrations used in this study. The dashed line indicates the ΔCT = 1.0 threshold for defining substantial inhibition. The truncated RT V1 construct retains the polymerase and connection domains and lacks the RNase H domain. Data in panel C are presented as mean ± SD from two biologically independent experiments, each performed with triplicate reactions.

To rationalize the resistance profiles observed in the benchmarking assays and in the saliva matrix proof-of-concept, we next examined the structural and mutational determinants associated with inhibitor tolerance. In this study, full-length RT V1 and RT V4 not only exhibited the strongest biochemical performance but also showed the most consistent resistance across a diverse panel of clinically relevant inhibitors. These two variants share five non-RNase H mutations, namely E69K, E302K/R, W313F, L435G, and N454K, which may act synergistically to enhance enzyme resilience. Interestingly, the same set of five mutations is also present in RT V3, which demonstrated strong inhibitor tolerance at 40 °C but reduced performance at elevated temperatures. This suggests that additional mutations unique to RT V3 may compromise stability under thermal stress, highlighting the importance of mutational context in defining enzyme robustness. The recurrence of this five-mutation combination in RT V1, V3, and V4, which were selected from over 30 tested substitutions, points to a recurrent mutational combination associated with inhibitor resistance. These mutations span key regions of the polymerase, including the fingers, thumb, and connection domain (Figure 8A), and have previously been linked to increased thermostability through tighter template–primer binding and improved tolerance to chemical inhibitors [18,21].

Structural analysis supports direct nucleic acid contact contributions for E69K, E302K/R, and N454K, each of which introduces additional positive charge and/or hydrogen-bonding capacity at the template–primer interface (Table S1) [36]. In contrast, W313F is not expected to create new polar contacts. Instead, W313 lies within a thumb-region surface implicated in minor-groove engagement, and prior mutational analyses in this region have proposed that phenylalanine substitutions can favor van der Waals contacts and/or aromatic packing with nucleic acid ligands [18]. Consistent with this view, W313F was selected as the preferred replacement in high-temperature activity screens, even though its mechanistic effect is likely mediated by subtle changes in local packing and substrate positioning rather than direct hydrogen bonding [18]. Finally, L435G, located in the connection domain, is positioned on the opposite side of the substrate-binding surface and is therefore unlikely to make direct contact with the template–primer substrate (Figure 8A). The removal of an important hydrophobic contact between the side chains of L435 and F369, resulting from the L435G change, supports the role of this mutation in modulating the enzyme’s conformational flexibility and/or facilitating interdomain movements, potentially enhancing substrate recognition [10,11,12,36].

Given the structural role of the connection domain, we hypothesized that mutations in this domain might be less critical than those in the finger or palm domains. To test this, we generated three RT V1 reversion variants: RT V1_G435L (reversion of L435G), RT V1_K454N (reversion of N454K), and the double revertant RT V1_G435L/K454N. In parallel, we evaluated the contribution of the C-terminal RNase H domain by comparing full-length RT V1, which consistently displayed high inhibitor tolerance in earlier experiments, with a truncated variant retaining only the N-terminal and connection domains, including the five shared mutations. All variants were assessed at 40 °C under strong inhibitor stress, using the highest concentrations of NZYol and plasma (Figure 8B,C) that strongly inhibit the WT RT. In inhibitor-free reactions, all constructs displayed closely matched amplification profiles (Figure 8B), indicating that the reversions and truncation did not compromise baseline performance under these conditions. Reversion of either or both mutations, as well as C-terminal truncation, had minimal impact on resistance to plasma, indicating that in this plasma-containing condition, resistance is largely preserved across these architectural and connection-domain perturbations. In contrast, both truncation and amino acid reversions in the connection domain impaired resistance to NZYol, suggesting that overall enzyme integrity and interactions that stabilize the enzyme–nucleic acid complex become more critical under strongly denaturing conditions (Figure 8B,C). Although direct substrate-binding measurements were not performed in this study, the convergence of mutational recurrence, structural positioning, published biochemical data, and targeted reversion analysis supports a model in which strengthened enzyme–nucleic acid engagement contributes substantially to inhibitor resistance. Future quantitative binding measurements will be important for defining the individual contributions of each substitution to substrate affinity and for distinguishing direct binding effects from broader changes in enzyme conformation or stability.

Taken together, these findings support the view that L435G contributes to increased conformational flexibility, while E69K and E302K/R (with supporting contributions from W313F and N454K) strengthen enzyme–nucleic acid engagement and provide a molecular foundation for inhibitor resistance. Importantly, the preservation of resistance in all truncated and revertant variants in plasma-containing reactions (25% *v*/*v* plasma), a condition in which M-MuLV WT RT is highly susceptible (Figure 5), supports an important role for substrate interaction dynamics, rather than domain architecture alone, as being central to reliable RT performance in complex diagnostic matrices.

## 4. Conclusions

This study provides a systematic dissection of how reverse transcriptase responds to diagnostic inhibitors, an often overlooked yet critical barrier to reliable RT-qPCR performance. By comparing M-MuLV WT RT with five engineered variants originally optimized for thermostability, we identified two lead candidates, RT V1 and RT V4, that consistently resisted a broad spectrum of clinically relevant inhibitors. Both enzymes share five key mutations (E69K, E302K/R, W313F, N454K, and L435G). Structural and functional analyses indicate that resistance is primarily associated with strengthened enzyme–nucleic acid interactions, supported by residues positioned at the polymerase–substrate interface (notably E69K and E302K/R, with contributions from W313F and N454K), while L435G likely modulates conformational flexibility. Reversion and truncation analyses further support the central role of substrate engagement in sustaining cDNA synthesis under inhibitory stress.

A notable finding of this study is that inhibitor resistance was maximal at conventional reverse transcription temperatures (≈40 °C), despite preserved catalytic competence up to 70 °C. This suggests that, while thermal resilience supports activity at elevated temperature, increased thermal stress may weaken productive enzyme–substrate engagement and/or increase susceptibility to chemical inhibition. In contrast, at moderate temperature, the enhanced substrate affinity conferred by the shared mutation set appears to better stabilize the enzyme–nucleic acid complex in chemically challenging environments.

Together, these results clarify design principles for inhibitor-resistant RTs. Strengthening substrate interactions emerges as an effective strategy to improve inhibitor tolerance, particularly in workflows that involve crude or partially purified samples. Future work will extend these principles to target-specific one-step RT-qPCR formats and representative diagnostic matrices and will focus on engineering RTs that preserve inhibitor resilience at elevated temperatures (5070 °C) to support robust cDNA synthesis in more demanding diagnostic settings. Overall, this integrative study, combining structural analysis, protein engineering, and diagnostic benchmarking, lays a foundation for next-generation reverse transcriptase tailored for high-sensitivity RNA detection when inhibitor carryover is difficult to avoid.

## Figures and Tables

**Table 1:** Engineered M-MuLV reverse transcriptase variants, amino acid substitutions, and predicted functional effects.

Variant	Amino Acid Changes	Predicted Functional Role	Reference
V1	E69K, E302R, W313F, L435G, N454K, D524G, E562Q, D583N	High thermostability, enhanced template affinity, and improved processivity. Finger domain mutations promote template stabilization, while connection domain mutations (L435G, N454K) may contribute to inhibitor resistance. ^†^	Arezi & Hogrefe [18]
V2	L139P, D200N, T330P, D449A, N479D, H594K, L603R	Increased thermostability and processivity. L139P and D200N enhance enzyme stability and substrate binding. T330P stabilizes a DNA-contacting helix in the thumb domain. D449A and N479D reinforce primer–template binding. H594K and L603R support structural integrity.	Baranauskas et al. [15]
V3	A32V, E69K, L72R, E286R, E302R, W313F, W388R, L435G, N454K, D524G, E562Q, D583N	Balanced thermostability and template interaction dynamics. A32V, L72R, and W388R enhance structural stability. E69K, E302R, and E286R improve primer–template interactions. L435G and N454K may modulate polymerase function and resistance to inhibitors. ^†^	Baba et al. [20]
V4	P51L, S67R, E69K, T197A, H204R, N249D, E302K, F309N, W313F, T330P, L435G, N454K, D524G, D583N, H594Q, D653N, L671P	Highly thermostable and processive. E69K, W313F, and L435G enhance stability. E302K and T330P improve template binding. H204R promotes thermostability and activity. F309N modifies primer binding. Connection domain changes (L435G, N454K) may improve resistance to inhibitors. ^†^	Oscorbin et al. [19]
V5	L52P, Y64R, R116M, Y133A, K152M, Q190F, T197Q, H204R, V223H, M289L, T306K, F309N, D524G, E562Q, D583N	Designed for increased thermostability with possible compromises in fidelity and efficiency. Y64R, R116M, and Q190F may weaken template interactions. K152M and T197Q affect dNTP handling. F309N and T306K modulate primer binding. H204R contributes to hyperactivity. ^†^	This study

^†^ RNase H catalytic activity was inactivated in full-length constructs (D524G, E562Q, D583N) to prevent RNase H-mediated degradation of RNA during reverse transcription and to support accumulation of full-length cDNA.

## Data Availability

The original contributions presented in this study are included in the article/Supplementary Materials. Further inquiries can be directed to the corresponding author.

## Supplementary Materials

The following supporting information can be downloaded at https://www.mdpi.com/article/10.3390/diagnostics16121881/s1: Figure S1: SDS-PAGE analysis of the recombinant M-MuLV RT enzyme panel; Figure S2: Effect of priming strategy and enzyme quantity on cDNA synthesis across different temperatures; Figure S3: Defining testing concentrations of clinically relevant RT-qPCR inhibitors through preliminary testing with RT V1; Figure S4: Evaluation of the effect of a clinically relevant panel of inhibitors on qPCR amplification; Figure S5: RNase contamination in clinically derived inhibitors and the protective effect of ribonuclease inhibitor; Figure S6: Assessment of DNase activity in clinically relevant inhibitors via hybrid cDNA degradation; Figure S7: Magnified view of inhibitor resistance among engineered full-length M-MuLV RT variants at 40 °C; Figure S8: Inhibitor resistance of engineered RT variants at elevated cDNA synthesis temperatures using the highest inhibitor concentrations; Table S1: Structural and biochemical roles of amino acid in engineered M-MuLV RT variants; Table S2: Clinically relevant inhibitors and their final test concentrations; Table S3: Paired RT-qPCR results for direct saliva testing and matched extraction-based testing in the spiked-saliva proof-of-concept study.

## Abbreviations

The following abbreviations are used in this manuscript:

RT	Reverse Transcriptase
RT-qPCR	Reverse Transcription Quantitative Polymerase Chain Reaction
RNA	Ribonucleic Acid
cDNA	Complementary DNA
M-MuLV	Moloney Murine Leukemia Virus
RNase H	Ribonuclease H
DNA	Deoxyribonucleic Acid
WT	Wild Type
PDB	Protein Data Bank
MAFFT	Multiple Alignment using Fast Fourier Transform
ESPript	Easy Sequencing in PostScript
CAI	Codon Adaptation Index
*E. coli*	*Escherichia coli*
IPTG	Isopropyl β-D-1-thiogalactopyranoside
IMAC	Immobilized Metal Ion Affinity Chromatography
SDS-PAGE	Sodium Dodecyl Sulfate Polyacrylamide Gel Electrophoresis
OD600	Optical Density at 600 nm
dNTPs	Deoxynucleotide Triphosphates
RI	Ribonuclease Inhibitor
qPCR	Quantitative Polymerase Chain Reaction
CT	Cycle Threshold
ΔCT	Difference in Cycle Threshold
FAM	Fluorescein Amidite
ROX	Carboxy-X-rhodamine
Rpl27	Ribosomal Protein L27
EDTA	Ethylenediaminetetraacetic Acid
K-EDTA	Potassium Ethylenediaminetetraacetic Acid
DNase	Deoxyribonuclease
RNase	Ribonuclease
FL	Full-Length
tr	Truncated
XMRV	Xenotropic Murine Leukemia Virus-related Virus
r.m.s.d.	Root Mean Square Deviation
SARS-CoV-2	Severe Acute Respiratory Syndrome Coronavirus 2
RdRp	RNA-dependent RNA Polymerase
IVD	In Vitro Diagnostic
*v*/*v*	Volume per Volume
ΔRn	Normalized Reporter Signal
LB	Luria–Bertani
