# Supplementary material for: Substrate Recognition Governs Reverse Transcriptase Resistance to Diagnostic Inhibitors in RT-qPCR
Source: Diagnostics (Basel). 2026 Jun 17;16(12):1881. doi: 10.3390/diagnostics16121881 (PMC13298206; doi:10.3390/diagnostics16121881)
Supplement: Supplementary file 1 [file diagnostics-16-01881-s001.zip › diagnostics-4266776-supplementary.pdf]

diagnostics

MDPI

Supplementary Information

Substrate Recognition Governs Reverse Transcriptase Resistance to Diagnostic Inhibitors in RT-qPCR

Inês F. Costa [1,2,3], Vânia O. Fernandes [1], Victor D. Alves [2,3], Virgínia M. R. Pires [1], Joana A. Brás [1], Pedro Bule [2,3,*] and Carlos M. G. A. Fontes [1,2,3]

1 NZYtech—Genes & Enzymes, Campus do Lumiar, Building J, 1649-038 Lisbon, Portugal; ines.costa@nzytech.com (I.F.C.); vania.fernandes@nzytech.com (V.O.F.); virginia.pires@nzytech.com (V.M.R.P.); joana.bras@nzytech.com (J.A.B.); carlos.fontes@nzytech.com (C.M.G.A.F.)

2 CIISA—Centre for Interdisciplinary Research in Animal Health, Faculty of Veterinary Medicine, University of Lisbon, 1300-477 Lisbon, Portugal; vdalves@fmv.ulisboa.pt

3 Associate Laboratory for Animal and Veterinary Sciences (AL4AnimalS), 1300-477 Lisbon, Portugal

* Correspondence: pedrobule@fmv.ulisboa.pt

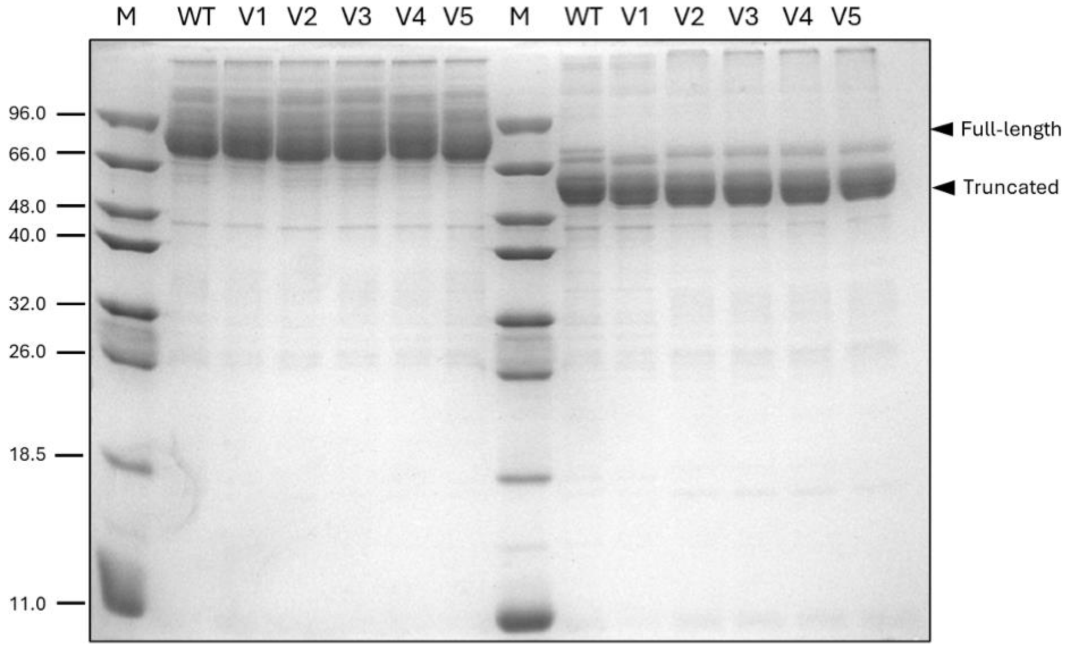

Figure S1. SDS-PAGE analysis of the recombinant M-MuLV RT enzyme panel. Coomassie-stained SDS PAGE gel showing soluble expression of twelve purified recombinant M-MuLV RT enzymes. Lanes correspond to wild-type (WT) and five engineered variants (V1–V5), each expressed in two forms: full-length and RNase H-inactive truncated versions. All proteins exhibit the expected molecular weight (~75 kDa for full-length and ~63 kDa for truncated proteins) and high solubility, confirming successful recombinant production and purification.

A

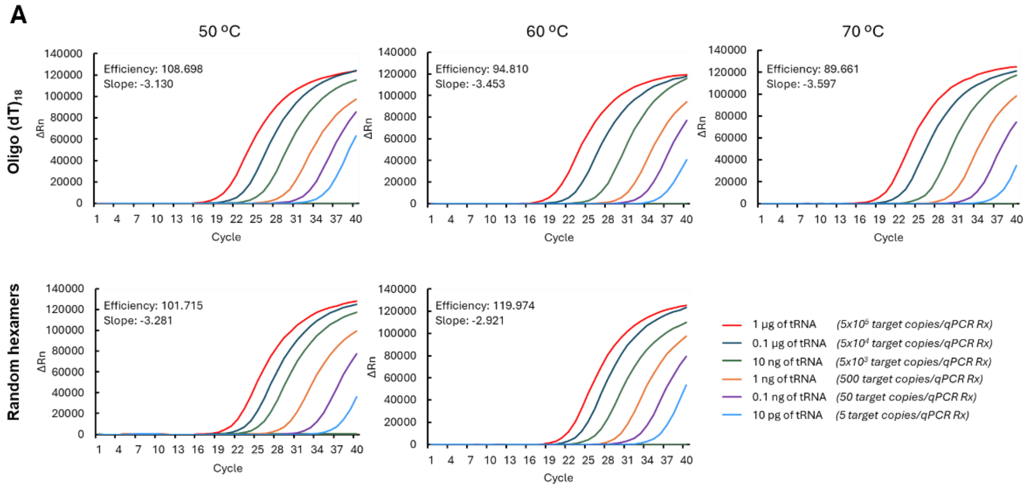

B

	Concentration	Priming	Eff %
50 °C	0.6 μg	Oligo(dT)$_{18}$	110.574
		Random hexamers	111.825
	0.7 μg	Oligo(dT)$_{18}$	108.698
		Random hexamers	101.715
	0.8 μg	Oligo(dT)$_{18}$	96.459
		Random hexamers	88.901
	0.9 μg	Oligo(dT)$_{18}$	91.965
		Random hexamers	86.962
60 °C	0.6 μg	Oligo(dT)$_{18}$	94.473
		Random hexamers	120.451
	0.7 μg	Oligo(dT)$_{18}$	94.810
		Random hexamers	119.974
	0.8 μg	Oligo(dT)$_{18}$	92.723
		Random hexamers	100.928
	0.9 μg	Oligo(dT)$_{18}$	89.104
		Random hexamers	88.294
70 °C	0.6 μg	Oligo(dT)$_{18}$	96.952
	0.7 μg		89.661
	0.8 μg		88.403
	0.9 μg		74.407

Figure S2. Effect of priming strategy and enzyme quantity on cDNA synthesis across different temperatures. Reverse transcription reactions were performed using full-length RT V1 and mouse liver total RNA with either oligo(dT)$_{18}$ or random hexamer primers. Reactions were conducted at 50 °C, 60 °C, and 70 °C using three different enzyme concentrations: 0.6 to 0.9 μg per 20 μL reaction. cDNA synthesis was quantified by qPCR targeting the *Mus musculus Rpl27* gene. **(A)** Representative RT-qPCR amplification curves were obtained using 0.7 μg of RT enzyme per reaction. **(B)** Summary of cDNA synthesis efficiency across all tested enzyme quantities. Oligo(dT)-primed reactions yielded high and consistent performance across all temperatures and enzyme levels. In contrast, random hexamer-primed reactions showed a marked impairment at temperatures of 60 °C or higher. All reactions were performed in triplicate.

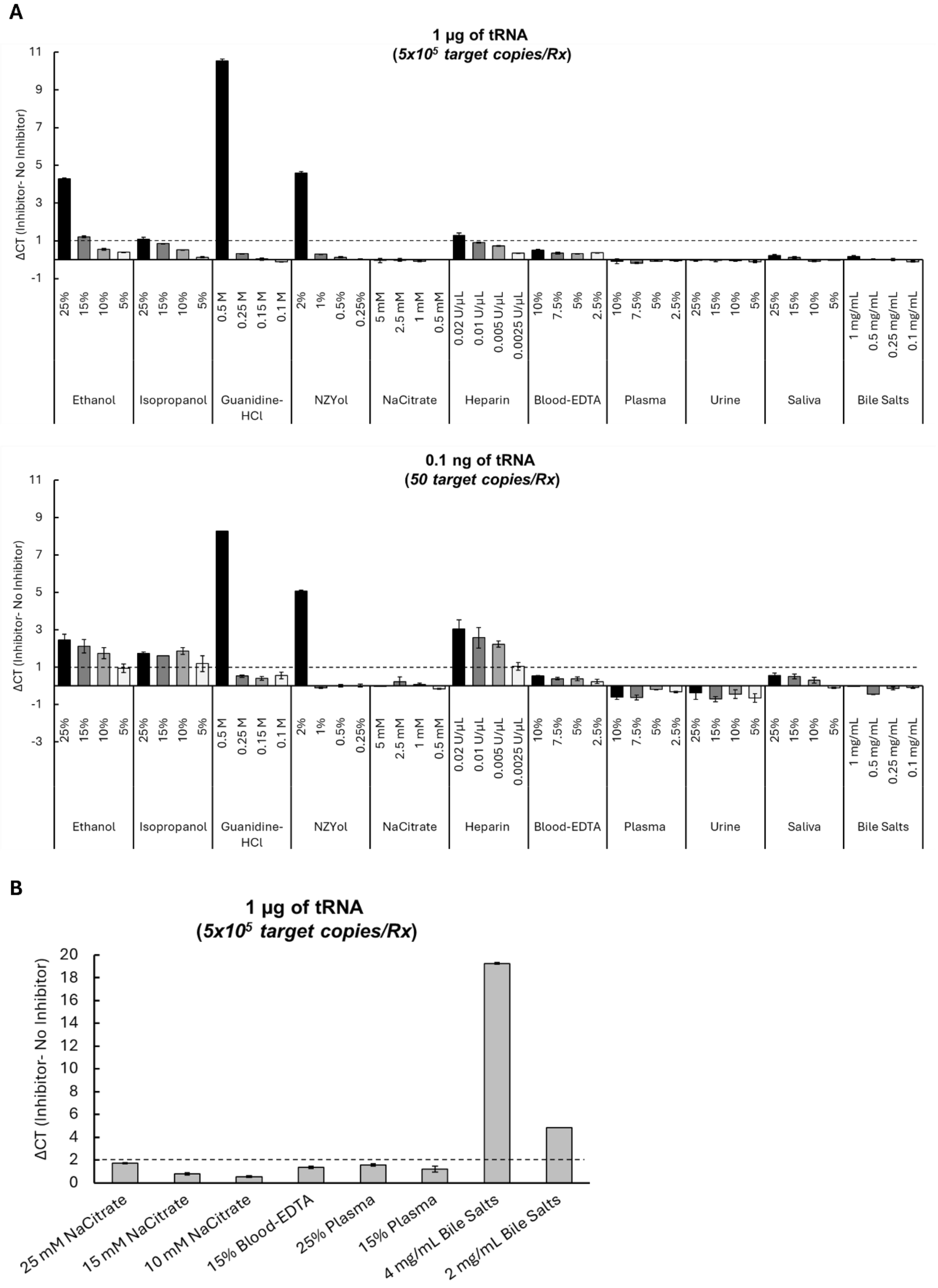

Figure S3. Defining testing concentrations of clinically relevant RT-qPCR inhibitors through preliminary testing with RT V1. (A) RT-qPCR ΔCT values derived from cDNA synthesized at 50 °C using RT V1 in the presence of eleven inhibitors at four different concentrations. Two RNA input levels (1 µg and 0.1 ng) were tested to simulate high and low target load conditions. **(B)** Selected inhibitors and selected concentrations were retested to better define inhibitory thresholds. The dashed line indicates the ΔCT = 1.0 threshold for defining substantial inhibition. Data are presented as mean ± SD from triplicate reactions.

A

	Inhibitor	[qPCR Conc]	ΔCT (Inhibitor- No inhibitor)
Nucleic acid extraction-related	Ethanol	2.5%	0.043
		1.5%	0.052
	Isopropanol	2.5%	-0.116
		1.5%	-0.183
	Guanidine-HCl	0.035 M	-0.250
		0.025 M	-0.092
	NZYol	0.2%	-0.095
		0.15%	-0.033
Biological matrix-related	NaCitrate	2.5 mM	0.946
		1.5 mM	0.341
	Heparin	0.002 U/µL	-0.159
		0.001 U/µL	-0.034
	Blood-EDTA	2.5%	0.343
		1.5%	0.183
	Plasma	2.5%	-0.302
		1.5%	-0.193
	Urine	2.5%	0.004
		1.5%	-0.068
	Saliva	2.5%	0.048
		1.5%	0.058
	Bile salts	0.3 mg/mL	0.089
		0.2 mg/mL	-0.138

B

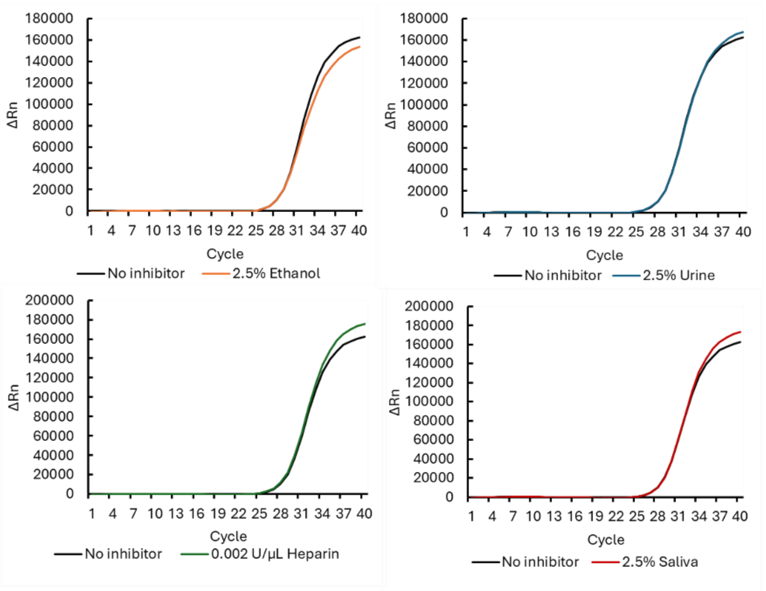

Figure S4. Evaluation of the effect of a clinically relevant panel of inhibitors on qPCR amplification. (A) Quantitative summary of ΔCT values (ΔCT is defined as the difference between the cycle threshold (CT) value of the reaction containing the inhibitor and that of the matched no-inhibitor control for each condition) is illustrated, indicating that qPCR amplification is generally unaffected by the defined inhibitor concentrations. *Mus musculus Rpl27* gene was amplified using 10 pg of pre-synthesized cDNA (assuming a 100% efficient conversion of 10 ng of total RNA). Calculated ΔCT values revealed that sodium citrate at 2.5 mM had the greatest impact on qPCR amplification. **(B)** Example of real-time amplification plots with 2.5 % ethanol, 0.002 U/µL heparin, 2.5% urine, and 2.5% saliva added directly to the qPCR reaction using cDNA synthesized in the absence of inhibitors. All reactions were performed in triplicate.

A

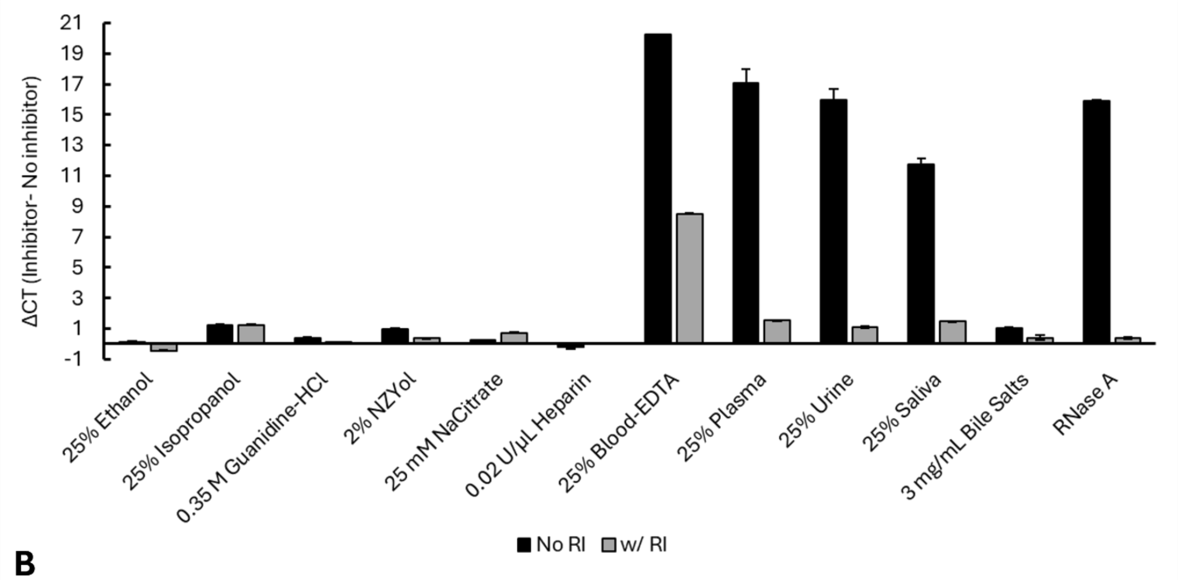

B

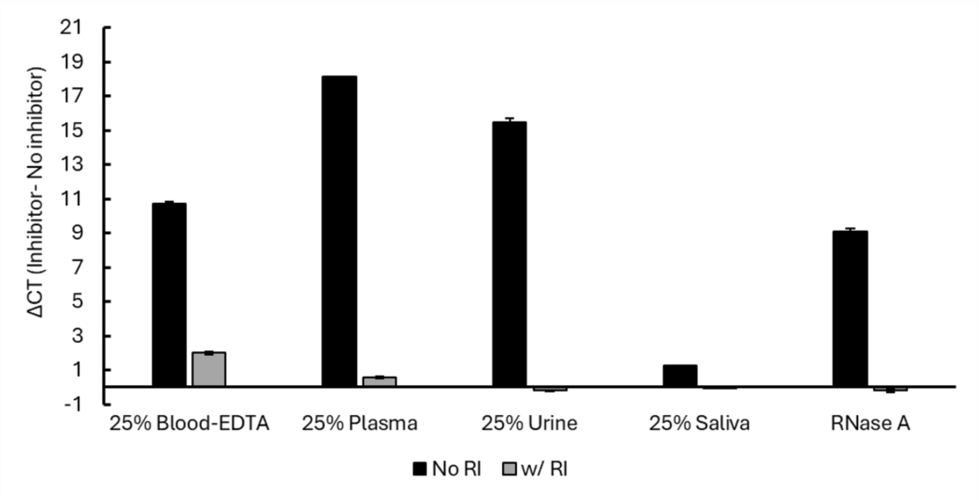

Figure S5. RNase contamination in clinically derived inhibitors and the protective effect of ribonuclease inhibitor. (A) Mouse liver total RNA (1 µg) was incubated for 20 min at 37 °C with each inhibitor at its maximum concentration, in the presence or absence of 40 U of NZY Ribonuclease Inhibitor (RI). Following incubation, RNA integrity was assessed using one-step RT-qPCR targeting the *Mus musculus Rpl27* gene. **(B)** To assess RNase contamination in the validated two-step workflow, cDNA synthesis reactions were performed with each inhibitor, with and without RI, followed by qPCR detection. The presence of RNase activity in blood-EDTA, plasma, urine, and saliva resulted in increased ΔCT values when RI was absent. RI-containing reactions preserved RNA integrity, confirming the effectiveness of RI under the tested conditions. A positive control for RNA degradation containing 5 ng of RNase A was included. Higher ΔCT values suggest RNA degradation. Data are presented as mean ± SD from triplicate reactions.

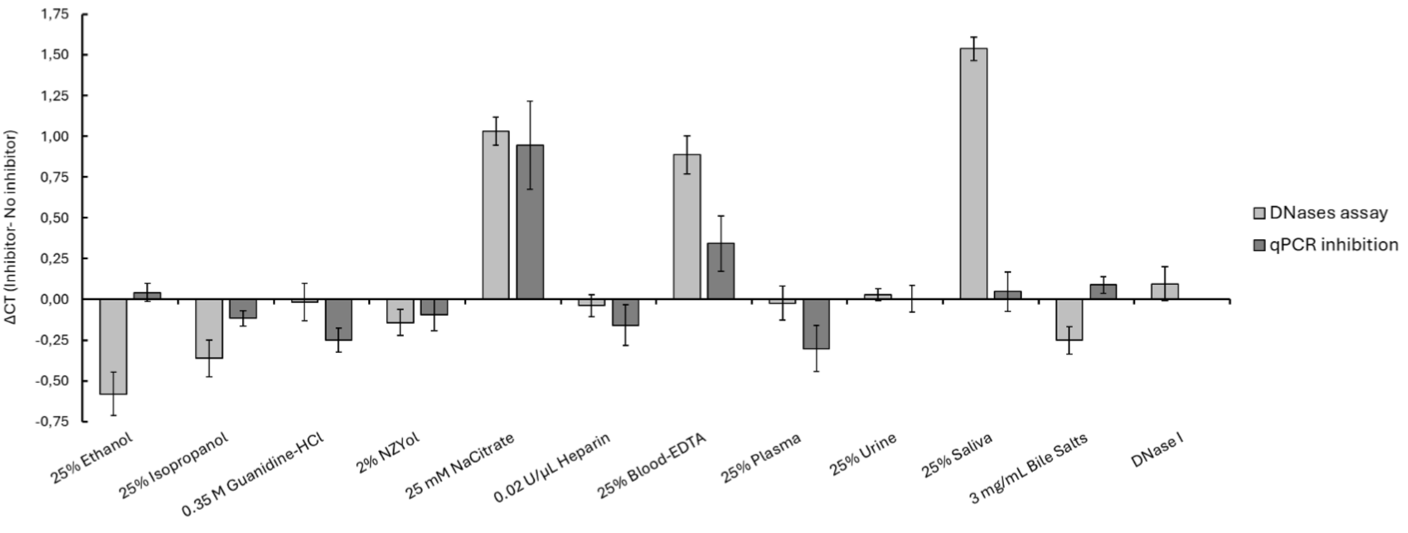

Figure S6. Assessment of DNase activity in clinically relevant inhibitors via hybrid cDNA degradation. Standard cDNA reactions using 0.01 ng of pre-synthesized cDNA as template were incubated with each inhibitor for 20 min at 37 °C. Results obtained in section 3.7 (qPCR inhibition) were included to control possible qPCR interference. ΔCT values reflect either cDNA degradation or qPCR inhibition. Saliva was not previously associated with qPCR inhibition (Figure S3), supporting the conclusion that the observed effect arises from DNase activity active at 37 °C. Data are presented as mean ± SD from triplicate reactions. All qPCR reactions were performed as described in Section 2.5.

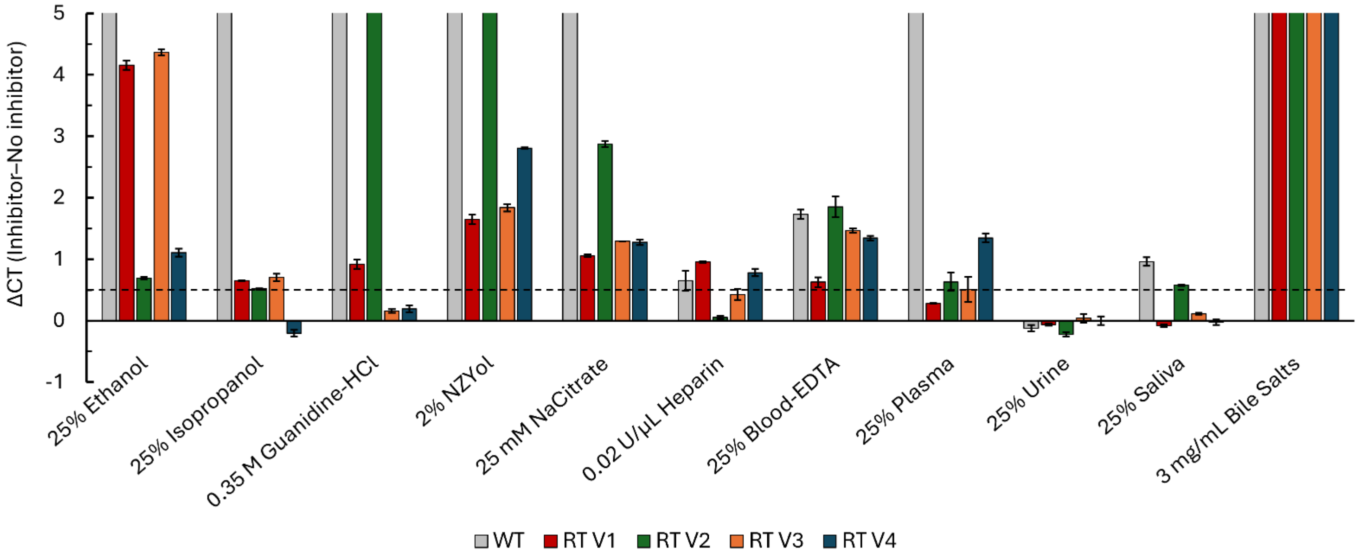

Figure S7. Magnified view of inhibitor resistance among engineered full-length M-MuLV RT variants at 40 °C. Zoomed representation of the lower ΔCT range from the dataset shown in Figure 5B, enabling clearer comparison among engineered RT variants V1–V4 in the presence of the highest tested concentrations of clinically relevant inhibitors. ΔCT values were calculated relative to matched no-inhibitor controls for each enzyme. Lower ΔCT values indicate higher resistance to inhibition, and the dashed line marks the predefined operational inhibition threshold (ΔCT = 1.0). Data are presented as mean ± SD from triplicate reactions.

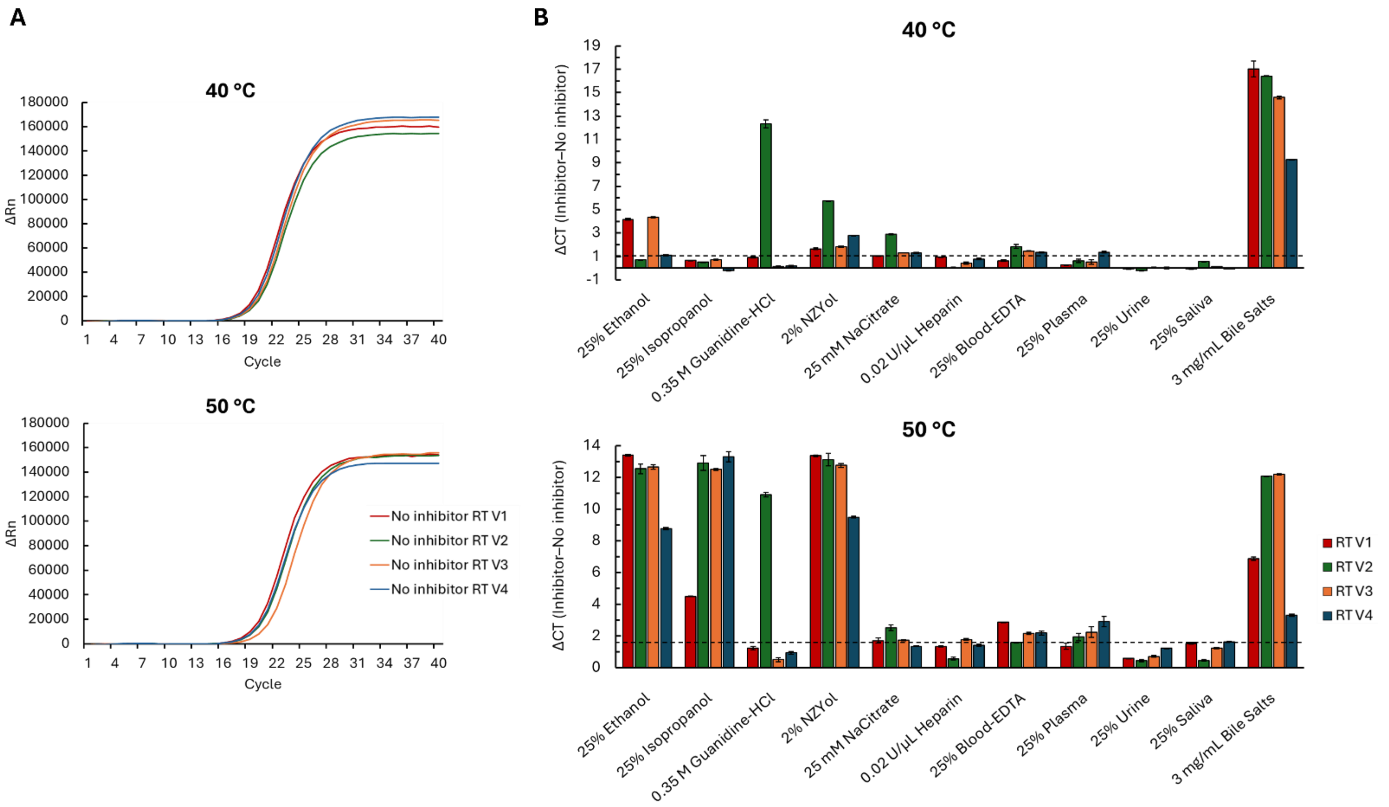

Figure S8. Inhibitor resistance of engineered RT variants at elevated cDNA synthesis temperatures using the highest inhibitor concentrations. **(A)** Real-time amplification curves obtained at 40 °C and 50 °C in the absence of inhibitors demonstrate that all four thermostable RT variants (RT V1–V4) exhibit consistent performance across these temperatures, confirming their robust intrinsic thermostability. **(B)** Bar plots show ΔCT values for each RT variant in the presence of the highest concentration of each inhibitor, calculated relative to no-inhibitor controls at 40 °C and 50 °C. The horizontal dashed line marks the defined inhibition threshold (ΔCT = 1.0). While inhibitor resistance remained strong at 40 °C, a sharp decline in tolerance was observed for nearly all inhibitor–enzyme combinations at 50 °C, with ΔCT values frequently exceeding the inhibition threshold of 1.0. Ethanol, isopropanol, NZYol, and Bile salts. Notably, bile salts exhibited reduced inhibition at 50 °C, possibly due to micellar disassembly at elevated temperature. These results indicate that the combined chemical and thermal stress significantly compromises RT activity, reinforcing 40 °C as the optimal cDNA synthesis temperature for diagnostic applications involving complex sample matrices. Data are presented as mean ± SD from triplicate reactions.

Table S1. Structural and biochemical roles of amino acid in engineered M-MuLV RT variants. The position (Pos) column denotes the amino acid residue number, while WT and Mut represent the wild-type and mutant amino acids, respectively. The Structural Role column describes the native function of each residue within the RT enzyme, while the Biochemical Role of mutant residue column highlights the predicted functional effects of the mutation.

Pos	WT	Structural Role	Mut	Biochemical Role of Mutant Residue
32	A	Hydrophobic pocket with L244, L247, A254	V	Thermostability; stabilization of hydrophobic core
51	P	Surface-exposed, not directly interacting with nucleic acids or core hydrophobic regions	L	Unknown
52	L	Hydrophobic interactions with L94 and L167; weak stacking with W94; surface	P	Increased thermostability
64	Y	Binds RNA template; stacking interactions with the base of +2 nucleotide (with L99)	R	Reduced fidelity, increased terminal transferase activity (weaker RNA template binding)
67	S	Surface-exposed, near E69, putatively binding DNA template	R	Unknown
69	E	Located at a solvent-exposed region near the template-RNA binding groove, but lacks direct nucleotide coordination	K	Higher affinity to template-RNA, increased thermostability due to substrate binding and efficient cDNA synthesis
72	L	Non-contacting surface residue	R	Increased thermostability; increases surface charge
116	R	Forms an electrostatic interaction with the phosphate backbone of RNA +1 nt, stabilizing the primer-template duplex	M	Reduced processivity and fidelity; elevated terminal transferase activity
133	Y	Forms a stacking interaction with the ribose sugar at -4 nt; stabilizes the nucleic acid positioning	A	Reduced terminal transferase activity
139	L	Hydrophobic core residue with interactions involving L136, I218, L220, L227, A229, L273, L359	P	Increased thermostability, affinity to template, and processivity
152	K	Coordinates the entrance of dNTPs with R80, D153 and K258	M	Reduced terminal transferase activity; altered dNTP coordination
190	Q	Located near the catalytic pocket, potentially stabilizing the incoming dNTP	F	dTTP binding residue; Severely decreased processivity while retaining affinity to template
197	T	Weak hydrogen bonding with 2′-OH of +2 nt, contributing to RNA recognition	Q	Reduced terminal transferase activity; altered template recognition
			A	Reduced terminal transferase activity; altered template recognition
200	D	Internal residue with side chain hydrogen bonding the carbonyl of Thr128	N	Increased thermostability, affinity to template and processivity
204	H	Surface residue is distant from template and product contacts	R	Increased thermostability and hyperactivity; decreased terminal transferase activity
223	V	Hydrophobic residue positioned beneath the primer strand, possibly stabilizing primer-template binding	H	Increased processivity and decreased Km for dNTPs; reduced terminal transferase activity
286	E	Exposed residue that does not directly interact with the primer-template complex	R	Increased thermostability
289	M	Exposed residue that does not directly interact with the primer-template complex	L	Increased thermostability
302	E	Coordinates with R298, forming a stabilizing interaction with the phosphate of -5 nt of primer	R	Increased thermostability and template-primer binding via additional hydrogen bonds
			K	Increased thermostability and template-primer binding via additional hydrogen bonds
306	T	Hydrophobic interaction with primer sugar nt -4	K	Thermostability. K may bind directly with phosphate of nt-4
309	F	Strong hydrophobic interaction with primer sugar at -3 nt	N	Decreased fidelity; elevated terminal transferase activity; altered primer-template interactions
313	W	Located in the thumb, forming part of the structural framework, stabilizing DNA binding	F	Increased thermostability, optimal temperature, and affinity to template
330	T	Disordered; may contact template RNA	P	Stabilization of DNA-contacting helical motif; increased thermostability and processivity
388	W	Solvent-exposed residue distal to the active site, no direct role in template binding	R	Increased thermostability via increased hydrophobic interactions

435	L	Hydrophobic contact with F369 and nearby residues, likely contributing to stability	G	Increased flexibility and thermostability
449	D	Located in a flexible region, possibly contributing to primer interactions or positioning	A	Improved affinity to template and thermostability
454	N	Located in a flexible region, possibly contributing to template-primer interactions	K	Increased thermostability
479	N	Exposed on the opposite side of the enzyme's nucleic acid-binding groove; unlikely to interact with template	D	Increased thermostability

Table S2. Clinically Relevant Inhibitors and their Final Test Concentrations. Summary of inhibitors selected to evaluate M-MuLV RT performance under clinically relevant conditions. The panel includes compounds commonly encountered during RNA extraction and those present in diagnostic specimens. Final test concentrations were selected based on literature reports and refined experimentally using RT V1 to define mild and stringent inhibition thresholds.

**Source**	**Inhibitor**	**Suggested concentrations in literature**	**Final concentrations used in this study**
RNA extraction	Ethanol	5–25% (v/v)	15% and 25% (v/v)
	Isopropanol	5–25% (v/v)	15% and 25% (v/v)
	Guanidine- Hydrochloride	0.1–0.5 M	0.25 M and 0.35 M
	NZYol	0.25–2% (v/v)	1.5% and 2% (v/v)
Clinical samples	Sodium Citrate	0.5-25 mM	15 mM and 25 mM
	Heparin	0.0025-0.02 U/µL	0.01 U/µL and 0.02 U/µL
	Whole Blood (EDTA-treated)	5–25% (v/v)	15% and 25% (v/v)
	Plasma	5–25% (v/v)	15% and 25% (v/v)
	Urine	5–25% (v/v)	15% and 25% (v/v)
	Saliva	5–25% (v/v)	15% and 25% (v/v)
	Bile Salts	0.1–4 mg/mL	2 mg/mL and 3 mg/mL

Table S3. Paired RT-qPCR results for direct saliva testing and matched extraction-based testing in the spiked-saliva proof-of-concept study. The table reports, for each sample, the SARS-CoV-2 Ct (RdRp/N channel), human RNase P internal control CT, and qualitative result obtained by one-step RT-qPCR performed directly on saliva (25% v/v input) or on RNA extracted from the matched saliva sample. Twenty SARS-CoV-2-negative clinical saliva specimens are included as specificity controls. Internal control amplification was used to validate negative results in accordance with the assay criteria. "Undetermined" indicates that no CT value was assigned under the assay conditions.

Sample Number	Saliva Pool	Direct RT-qPCR			RT-qPCR in Extracted Samples		
		SARS-CoV2 Ct	Internal control Ct	Result	SARS-CoV2 Ct	Internal control Ct	Result
1	1	18,82	25,42	Positive	18,97	27,91	Positive
2	1	21,49	26,92	Positive	21,57	27,63	Positive
3	1	23,83	29,56	Positive	23,90	27,81	Positive
4	1	26,18	30,33	Positive	26,39	28,11	Positive
5	1	28,30	29,98	Positive	28,44	28,60	Positive
6	1	30,87	27,30	Positive	30,65	28,14	Positive
7	1	30,94	30,45	Positive	31,87	29,08	Positive
8	1	31,55	30,97	Positive	31,69	30,62	Positive
9	2	16,44	24,63	Positive	17,31	24,72	Positive
10	2	18,98	23,81	Positive	19,97	24,45	Positive
11	2	21,04	25,77	Positive	21,27	26,31	Positive
12	2	23,92	25,28	Positive	23,47	25,72	Positive
13	2	21,86	24,10	Positive	23,31	25,12	Positive
14	2	19,51	23,56	Positive	19,49	24,48	Positive
15	2	20,99	26,59	Positive	20,80	26,31	Positive
16	2	24,13	26,13	Positive	24,51	26,27	Positive
17	3	21,19	28,03	Positive	20,89	28,59	Positive
18	3	26,34	30,79	Positive	25,69	28,64	Positive
19	3	30,30	31,52	Positive	27,91	28,98	Positive
20	3	22,80	27,61	Positive	23,34	30,71	Positive
21	3	25,93	30,88	Positive	28,06	29,82	Positive
22	3	18,00	25,39	Positive	18,64	29,66	Positive
23	3	20,57	28,92	Positive	21,42	31,04	Positive
24	3	23,48	26,96	Positive	23,25	32,74	Positive
25	4	25,69	25,34	Positive	26,42	29,07	Positive
26	4	28,44	28,57	Positive	28,28	28,59	Positive
27	4	31,15	26,72	Positive	30,35	30,85	Positive
28	4	34,61	28,42	Positive	32,70	29,17	Positive
29	4	21,88	22,42	Positive	19,43	26,65	Positive
30	4	32,84	28,38	Positive	29,95	27,29	Positive
31	4	31,73	24,16	Positive	Undetermined	Undetermined	*Inconclusive*
32	4	34,78	28,41	Positive	33,69	29,17	Positive
33		Undetermined	27,20	Negative	Undetermined	31,78	Negative
34		Undetermined	27,98	Negative	Undetermined	28,33	Negative
35		Undetermined	27,62	Negative	Undetermined	27,16	Negative
36		Undetermined	30,15	Negative	Undetermined	29,37	Negative
37		Undetermined	28,24	Negative	Undetermined	27,53	Negative
38		Undetermined	27,57	Negative	Undetermined	26,78	Negative
39		Undetermined	28,59	Negative	Undetermined	27,36	Negative
40		Undetermined	27,10	Negative	Undetermined	25,49	Negative
41		Undetermined	29,81	Negative	Undetermined	27,82	Negative
42		Undetermined	25,55	Negative	Undetermined	22,97	Negative
43		Undetermined	30,60	Negative	Undetermined	31,32	Negative
44		Undetermined	31,66	Negative	Undetermined	28,59	Negative
45		Undetermined	28,16	Negative	Undetermined	27,30	Negative
46		Undetermined	28,08	Negative	Undetermined	27,85	Negative
47		Undetermined	30,91	Negative	Undetermined	27,13	Negative
48		Undetermined	26,75	Negative	Undetermined	27,35	Negative
49		Undetermined	28,50	Negative	Undetermined	29,18	Negative
50		Undetermined	25,75	Negative	Undetermined	26,14	Negative
51		Undetermined	28,58	Negative	Undetermined	26,76	Negative
52		Undetermined	28,63	Negative	Undetermined	27,55	Negative
